# BuZhong YiQi Formula Alleviates Postprandial Hyperglycemia in T2DM Rats by Inhibiting α-Amylase and α-Glucosidase In Vitro and In Vivo

**DOI:** 10.3390/ph18020201

**Published:** 2025-02-02

**Authors:** Xin-Xin Zeng, Liang Wang, Ming-Yu Wang, Zhen-Ran Hu, Xiang-Ke Li, Guo-Jun Fei, Ling Ling, Yu-Ting Fan, Ze-Min Yang

**Affiliations:** 1 Department of Biochemistry and Molecular Biology, School of Basic Medical Sciences, Guangdong Pharmaceutical University, Guangzhou 510006, China; zengxinxin27@163.com (X.-X.Z.); liang7982023@163.com (L.W.); wmy112222@163.com (M.-Y.W.); 2112255029@stu.gdpu.edu.cn (Z.-R.H.); kekeli123123@163.com (X.-K.L.); feiguojun2024@163.com (G.-J.F.); lingl6963@163.com (L.L.); 2112255030@stu.dgpu.edu.cn (Y.-T.F.); 2 Guangdong Provincial Key Laboratory of Pharmaceutical Bioactive Substances, 280 Waihuan Road East in Guangzhou Higher Education Mega Center, Guangzhou 510006, China

**Keywords:** postprandial glycemic responses, enzyme kinetics, simulated digestion, total flavonoids, molecular docking

## Abstract

**Background/Objectives:** BuZhong YiQi Formula (BZYQF) can alleviate type 2 diabetes mellitus (T2DM). However, its efficacy in managing postprandial hyperglycemia in T2DM needs to be further confirmed, and its underlying mechanism and pharmacodynamic material basis have not been sufficiently investigated. **Methods:** A T2DM rat model was induced to measure postprandial glycemic responses following glucose and starch ingestion. In vitro assays of enzymatic inhibition and the kinetic mode were performed to evaluate the inhibitory effect of BZYQF on α-amylase and α-glucosidase activities. The main constituent contents of BZYQF in a simulated digestion assay were measured to screen the active constituents in BZYQF against α-amylase and α-glucosidase activities via Pearson correlation and multiple linear regression analyses. Finally, the total flavonoids were purified from BZYQF to perform in vitro activity validation, and the flavonoid constituent activity was verified through molecular docking. **Results:** In vivo assays showed that BZYQF significantly reduced the blood glucose values of CON rats but not T2DM rats after glucose ingestion, while BZYQF significantly reduced the blood glucose levels by 15 min after starch ingestion in CON and T2DM rats, with more significant decreases in blood glucose levels in T2DM rats. In vitro enzymatic assays showed that BZYQF could inhibit the activities of α-amylase and α-glucosidase in competitive and non-competitive modes and in an uncompetitive mode, respectively. Furthermore, BZYQF showed a stronger inhibitory effect on α-glucosidase activity than on α-amylase activity. Simulated digestion showed that simulated gastric fluid and intestinal fluid changed the main constituent contents of BZYQF and their inhibition rates against α-amylase and α-glucosidase activities, and similar results were rarely found in simulated salivary fluid. Pearson correlation and multiple linear regression analyses revealed that the total flavonoids were the active constituents in BZYQF inhibiting α-amylase and α-glycosidase activities. This result was verified by examining the total flavonoids purified from BZYQF. A total of 1909 compounds were identified in BZYQF using UPLC-MS/MS, among which flavones were the most abundant and consisted of 467 flavonoids. Molecular docking showed that flavonoids in BZYQF were bound to the active site of α-amylase, while they were bound to the inactive site of α-glucosidase. This result supported the results of the enzyme kinetic assay. **Conclusions:** BZYQF significantly alleviated postprandial hyperglycemia in T2DM rats by inhibiting α-amylase and α-glycosidase activities, in which flavonoids in BZYQF were the active constituents.

## 1. Introduction

Type 2 diabetes mellitus (T2DM) is caused by insufficient insulin and peripheral insulin resistance, resulting in hyperglycemia [1]. The International Diabetes Federation projections outline that approximately 783 million people will be living with T2DM by the year 2045. In addition, T2DM often leads to several complications, including nephropathy, retinopathy, neuropathy and cardiovascular diseases, which are the major causes of mortality in T2DM patients [2]. Epidemiological studies have suggested that postprandial hyperglycemia may be an independent risk factor of cardiovascular disease and mortality in T2DM patients [3]. Therefore, it is particularly important for T2DM patients to reduce postprandial hyperglycemia and maintain stable blood glucose levels.

The theory of traditional Chinese medicine (TCM) considers that T2DM belongs to the category of “Xiaoke disease”, with its main cause being spleen and stomach deficiency. As a TCM term, spleen and stomach deficiency refers to the impairment of the patient’s spleen and stomach functions, manifested by weakened digestion, absorption, and metabolism of nutrients. The latest evidence shows that TCMs, including Chinese traditional patent medicines, empirical formulas, and monomers, not only demonstrate clinical efficacy comparable to that of existing Western drugs in managing hypoglycemia but also offer fewer adverse effects and a multitarget therapeutic approach [4]. BuZhong YiQi Formula (BZYQF) is a classic formula for treating spleen and stomach deficiency. BZYQF is composed of eight Chinese herbs: *Astragalus membranaceus*, *Glycyrhiza uralensis*, *Ginseng quinquefolium*, *Atractylodes macrocephala*, *Angelica sinensis*, *Cimicifuga heracleifolia*, *Bupleurum chinense*, and *Citrus reticulata*. Modern pharmacological studies have found that all the herbs *Astragalus membranaceus*, *Ginseng quinquefolium*, *Atractylodes macrocephala*, and *Citrus reticulata*, as well as their constituents in BZYQF, exhibit hypoglycemic effects [5,6,7,8]. Clinical observations have found that BZYQF could significantly improve fasting blood glucose, Hb A1c, and 2 h postprandial blood glucose in patients with T2DM [9]. These studies have revealed the hypoglycemic effects of BZYQF in T2DM rats and patients. However, these studies have mainly focused on fasting blood glucose, with limited reports on the role of BZYQF in managing postprandial hyperglycemia in T2DM.

Postprandial blood glucose generally refers to the blood glucose level measured 1 or 2 h after a meal. In this process, α-amylase in the salivary glands and pancreas hydrolyses dietary starch into maltose, maltotriose, isomaltose, and α-limit dextrin, which consists of four to nine glucose residues. Subsequently, α-glucosidase in intestinal mucosa hydrolyses maltose and maltotriose into glucose. The released glucose is absorbed into the bloodstream by the transporters of SGLT-1 and GLUT2, resulting in an increase in blood glucose levels. Therefore, the inhibition of α-amylase and α-glucosidase activities prevents a sudden surge in glucose, leading to lower postprandial hyperglycemia [10]. Acarbose, a drug that inhibits α-amylase and α-glucosidase inhibitors, has been widely used to control postprandial hyperglycemia in T2DM patients. However, acarbose causes gastrointestinal side effects, including diarrhea, abdominal pain, and flatulence [11]. Therefore, it is urgent to discover novel α-amylase and α-glucosidase inhibitors with fewer side effects.

In this study, we characterized the chemical constituents in BZYQF using UPLC-MS/MS analysis and evaluated the effect of BZYQF on postprandial hyperglycemia in T2DM rats. Subsequently, we investigated the inhibitory effect of BZYQF on α-amylase and α-glucosidase activities in vitro. Finally, we measured the main constituent contents of BZYQF during simulated digestion and screened the active constituents in BZYQF that inhibit α-amylase and α-glycosidase activities using Pearson’s correlation analysis, multiple linear regression analysis, and molecular docking. The present study provides experimental evidence for the treatment of T2DM with BZYQF through the inhibition of α-amylase and α-glucosidase activities.

## 2. Results

### 2.1. Chemical Constituents of BZYQF

**Figure 1:**
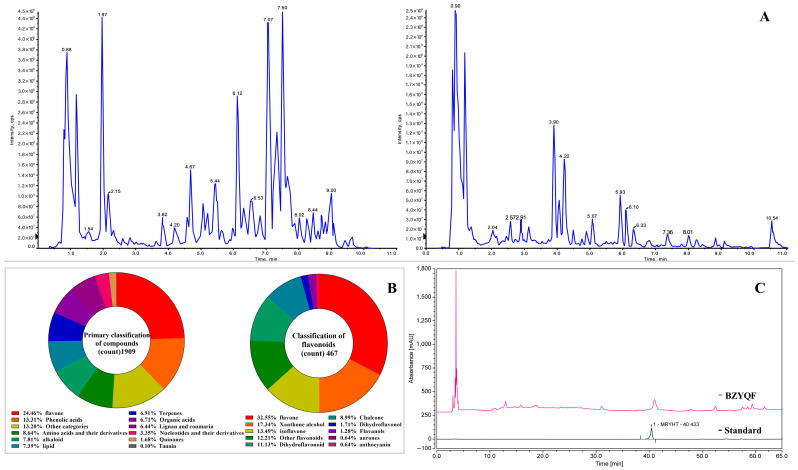
Chemical constituent characterization of BZYQF. (**A**) Total Ion Chromatograms of positive ion mode (left) and negative ion mode (right); (**B**) Primary and secondary classification of compounds identified in BZYQF; (**C**) HPLC chart for determination of calycosin-7-O-β-D-glucoside (MRYHT) content in BZYQF.

A total of 1909 primary compounds were identified in BZYQF through a widely targeted metabolic profiling analysis (Figure 1 and Supplementary Material). The primary compounds accounting for more than 5% were flavones, phenolic acids, amino acids, and their derivatives, alkaloids, lipids, terpenoids, organic acids, lignans, and coumarins. Among them, flavones had the highest proportion at almost 25% and consisted of 467 secondary flavonoids. These flavonoids mainly included flavones, xanthone alcohols, isoflavones, dihydroflavonoids, chalcones, and other flavonoids. The content of calycosin-7-O-β-D-glucoside, an isoflavone from *Astragalus membranaceus*, was 0.83 mg/g pills, which was the highest content among the flavonoids in BZYQF (Supplementary Material).

The Qquantification of the main constituents in BZYQF revealed that the total carbohydrate content was 146.72 ± 2.59 mg/g; the reducing sugar content was 86.22 ± 1.11 mg/g; the polysaccharide content was 60.50 ± 3.12 mg/g; the total flavonoid content was 23.45 ± 4.39 mg/g; the total polyphenol content was 123.79 ± 2.68 mg/g; and the total saponin content was 8.11 ± 3.05 mg/g.

### 2.2. BZYQF Ameliorated Postprandial Hyperglycemia in T2DM Rats

**Figure 2:**
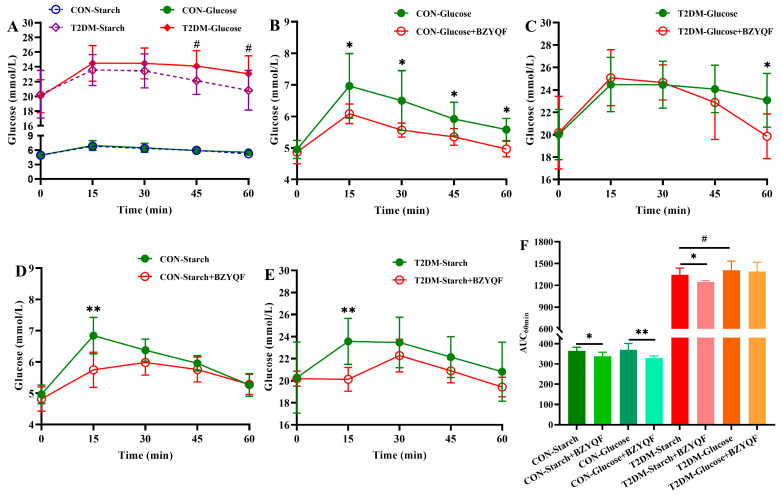
Blood glucose value and AUC of experimental rats after the ingestion of glucose and starch solutions. (**A**) Blood glucose value of experimental rats after the ingestion of glucose and starch solutions without BZYQF; (**B**) Blood glucose value of CON rats after the ingestion of glucose solution; (**C**) Blood glucose value of T2DM rats after the ingestion of glucose solution; (**D**) Blood glucose value of CON rats after the ingestion of starch solution; (**E**) Blood glucose value of T2DM rats after the ingestion of starch solution; (**F**) the AUC of experimental rats after the ingestion of glucose and starch solutions. * *p* < 0.05, ** *p* < 0.01, compared with starch or glucose solution without BZYQF; # *p* < 0.05, compared with glucose solution.

To evaluate the effect of BZYQF on postprandial hyperglycemia in T2DM rats, in vivo postprandial glycemic responses following starch and glucose ingestion were observed. The results of postprandial glycemic responses following the ingestion of starch and glucose solutions without BZYQF intervention are shown in Figure 2A,F. Following the ingestion of glucose and starch solutions, the blood glucose values of CON rats significantly increased and peaked at 15 min, followed by a rapid decline to near baseline level. Furthermore, the blood glucose values at any time point and AUC of CON rats did not differ between the ingestion of the two solutions. This finding reveals that both starch and glucose solutions could rapidly increase the blood glucose levels of CON rats, and there was no difference in glycemic response between the two solutions. Following the ingestion of glucose and starch solutions, the blood glucose values of T2DM rats significantly increased and peaked at 15 min, followed by a decline. Moreover, the blood glucose values of T2DM rats differed over time between the ingestion of the two solutions. Specifically, the blood glucose values of T2DM rats at 45 and 60 min after the ingestion of starch solution were significantly lower than those after the ingestion of glucose solution, and the AUC of the former was significantly lower than that of the latter. This finding reveals that both starch and glucose solutions could rapidly increase the blood glucose levels of T2DM rats, but the glycemic response of starch solution was lower than that of glucose solution. In addition, the blood glucose values and AUC of T2DM rats were significantly higher than those of CON rats after the ingestion of the two solutions. This result suggests that CON rats were able to rapidly digest starch and utilize glucose, thereby controlling blood glucose stability, while T2DM rats showed weakened glucose metabolism due to impaired starch digestion and glucose uptake.

The results of postprandial glycemic responses following the ingestion of glucose solution with BZYQF intervention are shown in Figure 2B,C,F. BZYQF significantly reduced the blood glucose values and AUC of CON rats at all time points after the ingestion of glucose solution, while BZYQF only significantly decreased the blood glucose value of T2DM rats at 60 min after the ingestion of glucose solution. These results suggest that BZYQF was able to better control the blood glucose values of CON rats to the baseline level within 60 min by regulating glucose metabolism (such as by promoting insulin secretion and glucose uptake). At the same time, due to impaired glucose metabolism in T2DM rats, BZYQF had no effect on their blood glucose values after the ingestion of glucose solution in a short period of time (within 45 min), but it might have a certain improvement effect after a prolonged intervention time. The results of postprandial glycemic responses following the ingestion of starch solution with BZYQF intervention are shown in Figure 2D–F. BZYQF significantly reduced the blood glucose values of CON and T2DM rats at 15 min, and delayed the peak of their blood glucose values from the 15 min to 30 min time point. BZYQF had no effect on the blood glucose values at other time points after the ingestion of starch solution. Furthermore, the AUC of CON and T2DM rats significantly decreased after BZYQF intervention. These results suggest that BZYQF reduced postprandial blood glucose levels in CON and T2DM rats at 15 min after the ingestion of starch solution by inhibiting starch hydrolysis. In addition, BZYQF caused a greater drop in blood glucose values at 15 min in T2DM rats than in CON rats (the delta blood glucose value decreased 2.02-fold from 1.88 to 0.93 in CON rats, and the delta blood glucose value reduced 9.65-fold from 3.28 to 0.34 in T2DM, *p* < 0.05). This finding indicates that after the ingestion of starch solution, BZYQF caused a more significant reduction in postprandial blood glucose levels in T2DM rats compared to CON rats.

### 2.3. BZYQF Inhibited α-Amylase and α-Glucosidase Activities In Vitro

**Figure 3:**
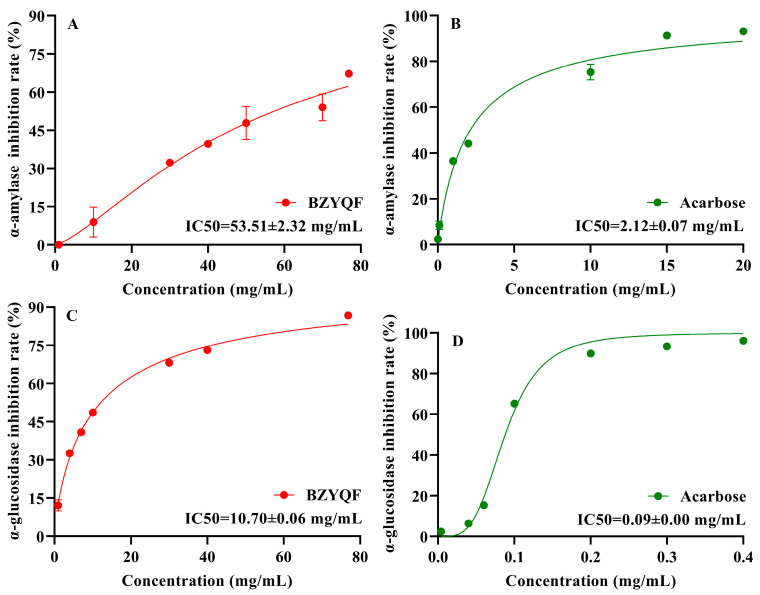
The IC50 values of BZYQF and acarbose for α-amylase and α-glucosidase inhibition. (**A**) the IC50 values of BZYQF for α-amylase inhibition; (**B**) the IC50 values of acarbose for α-amylase inhibition; (**C**) the IC50 values of BZYQF for α-glucosidase inhibition; (**D**) the IC50 values of acarbose for α-glucosidase inhibition.

To determine the inhibitory effect of BZYQF on the activities of α-amylase and α-glucosidase, in vitro enzyme inhibition assays were performed. The results are shown in Figure 3. The results demonstrate that the inhibitory effect of BZYQF on the activities of α-amylase and α-glucosidase steeply increased with an increase in its concentration, and then increased at a slower rate. The IC50 values of BZYQF against α-amylase and α-glucosidase activities were 53.51 ± 2.32 mg/mL and 10.70 ± 0.06 mg/mL, respectively. The IC50 values of acarbose against α-amylase and α-glucosidase were 2.12 ± 0.07 mg/mL and 0.09 ± 0.00 mg/mL, respectively. These results reveal that BZYQF and acarbose had notable inhibitory effects on the activities of α-amylase and α-glucosidase, and their inhibitory effects on the activity of α-glucosidase were stronger than those of α-amylase.

### 2.4. BZYQF Inhibited α-Amylase and α-Glucosidase Activities Through Different Kinetic Modes

**Figure 4:**
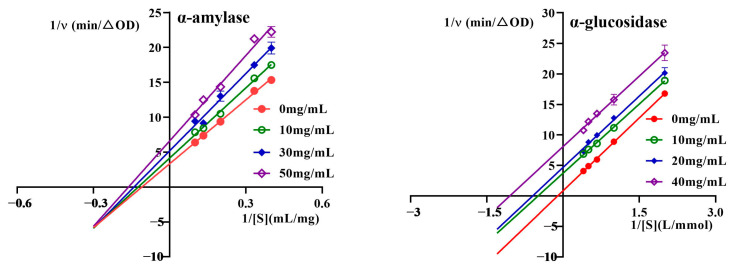
Lineweaver–Burk plots of α-amylase and α-glucosidase inhibitions by BZYQF.

To confirm the inhibition modes of α-amylase and α-glucosidase by BZYQF, the enzyme kinetic parameters, Km and Vmax, were determined using Lineweaver–Burk plots. The results are shown in Figure 4 and Table 1. For α-amylase, all lines fit linearly and intersect in the third quadrant. The values of Km and Vmax decrease simultaneously with an increase in the BZYQF concentration, while the slope of the straight line increases from 30.53 to 40.81, indicating that BZYQF could bind to α-amylase in mixed competition mode. This mode means that some compounds in BZYQF could bind to active sites of α-amylase in a competitive mode, while others bound to the inactive sites of α-amylase in a non-competitive mode. For α-glucosidase, all lines fit linearly and are parallel. The values of Km and Vmax decrease simultaneously with an increase in the BZYQF concentration, while the slope of the straight line remains almost constant, indicating that BZYQF could bind to an α-glucosidase–substrate complex in an uncompetitive mode. These results suggest that BZYQF inhibited α-amylase and α-glucosidase activities in different kinetic modes.

### 2.5. In Vitro Simulated Digestion Changed the Main Constituent Contents of BZYQF and Their Inhibition Rates for α-Amylase and α-Glucosidase

**Figure 5:**
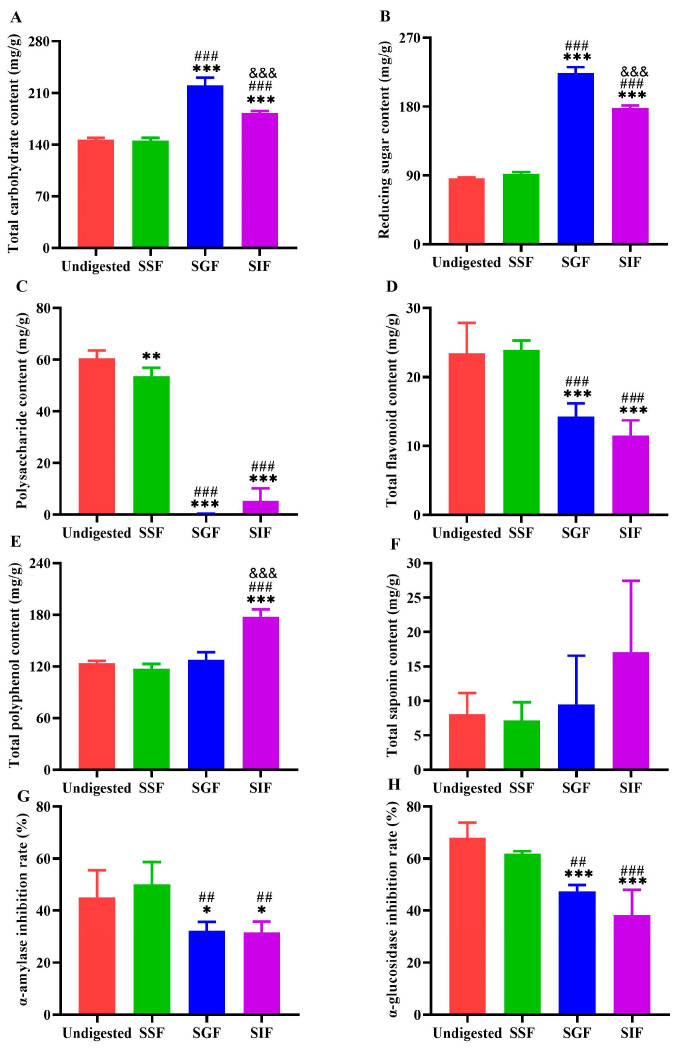
The main constituent contents of BZYQF and their inhibition rates for α-amylase and α-glucosidase during in vitro simulated digestion. (**A**) Total carbohydrate content; (**B**) Reducing sugar content; (**C**) Polysaccharide content; (**D**) Total flavonoid content; (**E**) Total polyphenol content; (**F**) Total saponin content; (**G**) the α-amylase inhibition rate; (**H**) the α-glucosidase inhibition rate. * *p* < 0.05, ** *p* < 0.01, *** *p* < 0.001, compared with undigested group; ## *p* < 0.01, ### *p* < 0.001, compared with SSF group; &&& *p* < 0.001, compared with SGF group.

To explore the influence of digestive fluids on BZYQF, in vitro simulated digestion was performed. The changes in the main constituent contents of BZYQF during in vitro simulated salivary–gastrointestinal digestion are shown in Figure 5A–F. The contents of total carbohydrate and reducing sugar demonstrated no significant change, while the polysaccharide content notably decreased during salivary digestion. Interestingly, the contents of total carbohydrate and reducing sugar significantly increased during gastric digestion, while their contents notably decreased after intestinal digestion. On the contrary, the polysaccharide content clearly reduced during gastric digestion but showed no significant change after intestinal digestion. The total flavonoid and total polyphenol contents demonstrated no significant change during salivary digestion. However, the total flavonoid content gradually decreased after gastrointestinal digestion, while the total polyphenol content markedly increased after intestinal digestion. In addition, the total saponin content demonstrated no significant change during salivary–gastrointestinal digestion. These results suggest that the main constituent contents of BZYQF were less affected by SSF, but their contents were greatly influenced by SGF and SIF.

The changes in the inhibition rates of α-amylase and α-glucosidase by BZYQF during in vitro simulated salivary–gastrointestinal digestion are shown in Figure 5G,H. The inhibition rates of α-amylase and α-glucosidase by BZYQF showed no significant change during salivary digestion. Interestingly, the inhibition rates of α-amylase and α-glucosidase significantly decreased during gastric digestion. These results suggest that the inhibition rates of α-amylase and α-glucosidase by BZYQF are closely related to its main constituent contents.

### 2.6. The Total Flavonoids Played an Important Role in the Inhibition of α-Amylase and α-Glucosidase Activities by BZYQF

**Figure 6:**
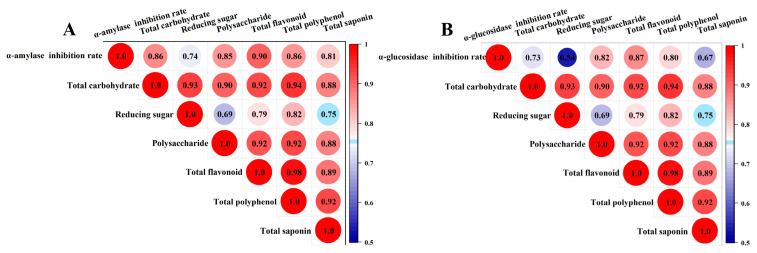
Pearson correlation coefficient (r) between the main constituent contents of BZYQF and their inhibition rates of α-amylase (**A**) or α-glucosidase (**B**).

To clarify the active constituents of BZYQF in the inhibition of α-amylase and α-glucosidase activities, we performed Pearson correlation analysis and multiple linear regression analysis between the inhibition rate of α-amylase and α-glucosidase and the main constituent contents of BZYQF. As illustrated in Figure 6, the total flavonoid content was highly correlated with the inhibition rates of α-amylase (*r* = 0.90) and α-glycosidase (*r* = 0.87), with the highest correlation coefficients among all factors. As listed in Table 2, the formula generated by the multiple linear regression analysis was as follows: the inhibition rate of α-amylase = 0.319 + 0.294 × total flavonoid and the inhibition rate of α-glucosidase = 0.532 + 0.236 × total flavonoid. The non-significant coefficients (*p* > 0.05) were removed from the analysis, showing that the response variables were only dependent on the factor of total flavonoids and were positively influenced by this factor. The factors of total carbohydrates, reducing sugar, polysaccharides, total polyphenols, and total saponins were not significant, showing no significant influence on the response variables. The *R^2^* values indicate that the variability in the inhibition rates of α-amylase and α-glycosidase could be explained by the fitting of the model, as their values are greater than 85% (85.0% and 85.6%, respectively). These results suggest that the total flavonoids might be the active constituents of BZYQF in the inhibition of α-amylase and α-glucosidase activities.

### 2.7. Purification of Total Flavonoids and In Vitro Activity Validation

**Figure 7:**
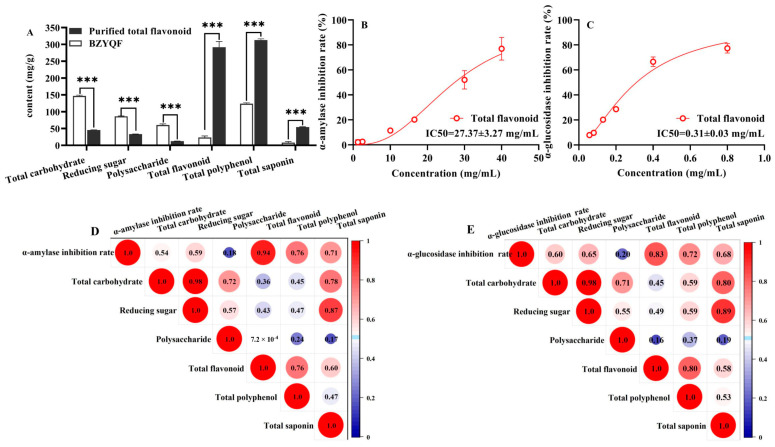
The main constituent contents, the IC50 values, and Pearson correlation coefficients of the total flavonoids purified from BZYQF. (**A**) The main constituent contents of BZYQF and the purified total flavonoids; (**B**,**C**) the IC50 values of the purified total flavonoids for α-amylase and α-glucosidase inhibition; (**D**,**E**) Pearson correlation coefficients between the main constituent contents of the purified total flavonoids and their inhibition rates of α-amylase (**D**) or α-glucosidase (**E**). *** *p* < 0.001, compared with BZYQF.

To verify the biological activity of the total flavonoids, they were extracted and isolated from BZYQF to measure the main constituent content and perform in vitro inhibition assays of α-amylase and α-glucosidase. As shown in Figure 7A, after the isolation of D101 macroporous resin, the total carbohydrate, reducing sugar, and polysaccharide contents significantly decreased, while the total flavonoid, total polyphenol, and total saponin contents remarkably increased in the purified total flavonoids compared with BZYQF. Furthermore, the total flavonoid content increased 12.44-fold from 23.45 to 291.94 mg/g crude drug (equivalent to 625.23 mg/g dry powder) with the most significant increase. After purification, flavonoids and polyphenols were the main constituents of the purified total flavonoids. As illustrated in Figure 7B,C, the IC50 values of the purified total flavonoids against α-amylase and α-glucosidase activities were 27.37 ± 3.27 mg/mL and 0.31 ± 0.03 mg/mL, respectively. As indicated in Figure 7D,E, in the purified total flavonoids, the total flavonoid content was highly correlated with the inhibition rates of α-amylase (*r* = 0.94) and α-glycosidase (*r* = 0.83), with the highest correlation coefficients among all factors. As listed in Table 3, in the purified total flavonoids, the formula generated from multiple linear regression analysis was as follows: the inhibition rate of α-amylase = 0.046 × total flavonoid and the inhibition rate of α-glucosidase = 0.219 + 0.051 × total flavonoid. Their *R^2^* values were greater than 80% (93.1% and 80.6%, respectively). These results suggest that the purified total flavonoid had stronger inhibitory effects on the activities of α-amylase and α-glucosidase than BZYQF, which is closely related to the increase in the total flavonoid content.

### 2.8. Molecular Docking of Flavonoids in BZYQF with α-Amylase and α-Glycosidase

**Figure 8:**
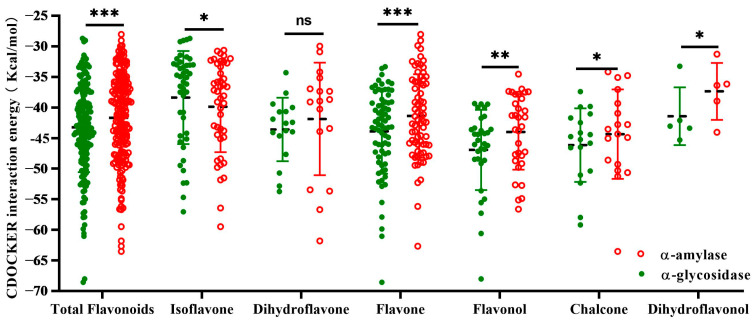
Interaction energy of flavonoids in BZYQF with α-amylase and α-glycosidase. * *p* < 0.05, ** *p* < 0.01, *** *p* < 0.001; ns, not statistically significant.

Molecular docking was performed to further understand the binding mechanism between flavonoids in BZYQF and α-amylase/α-glycosidase, and the associated structures were characterized using Discovery Studio. Out of the 467 flavonoids identified in BZYQF, 197 of them were able to bind to α-amylase or α-glycosidase. Among them, 187 flavonoids could bind to α-amylase and α-glycosidase simultaneously, including 5 dihydroflavonols, 18 chalcones, 32 flavonols, 71 flavones, 44 isoflavones, and 17 dihydroflavones. As presented in Figure 8, the binding of 187 flavonoids to the two enzymes was mainly characterized by low (interaction energy > −40) and medium (−60 < interaction energy < −40) binding intensities. Among them, the number and percentage of flavonoids with moderate binding intensity to α-glycosidase were significantly higher than those of flavonoids bound to α-amylase. In addition, the average interaction energy of all of these flavonoids to α-glucosidase was significantly lower than their average interaction energy to α-amylase. Similar results were found in other secondary flavonoids, with the exception of isoflavones, in which a low binding intensity predominated. These results suggest that flavonoids in BZYQF more strongly bound to α-glycosidase than to α-amylase.

**Figure 9:**
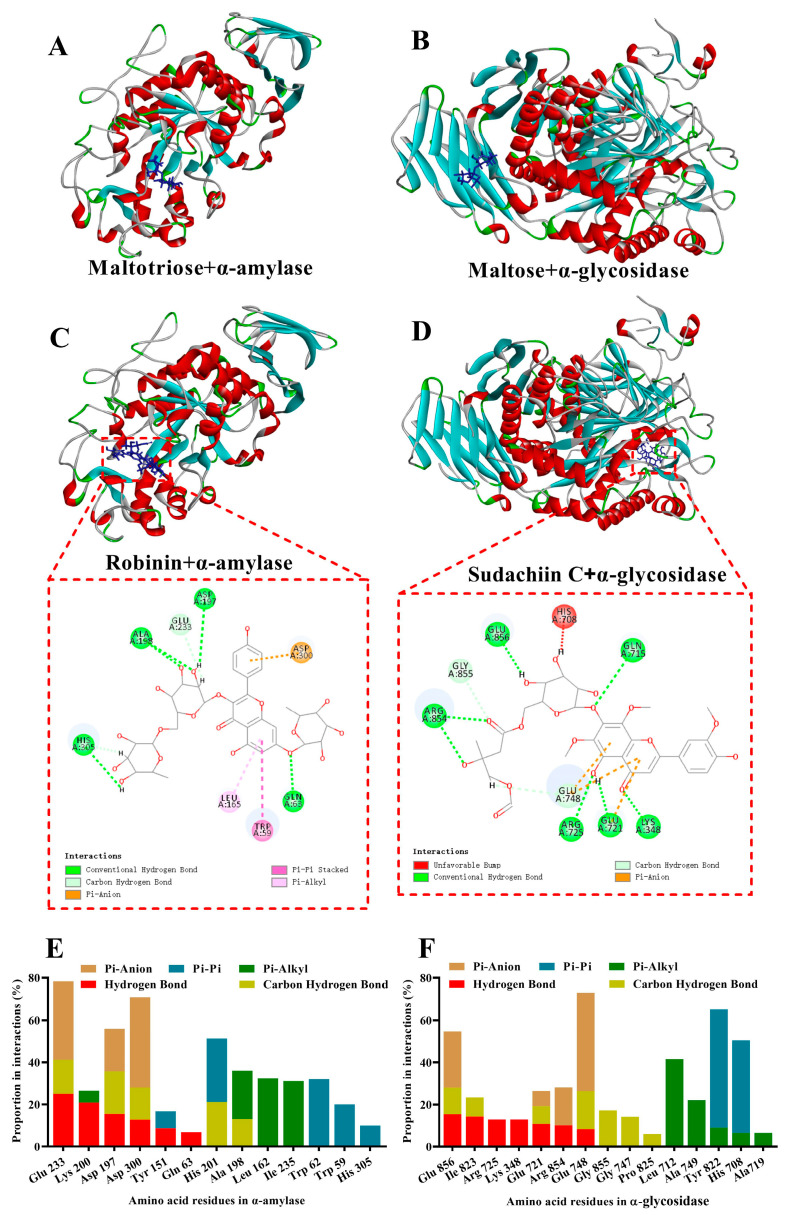
The docked diagrams and the proportion of amino acid residues in interactions based on the predicted optimal binding mode of flavonoids in BZYQF to α-amylase and α-glucosidase. (**A**,**B**) Three-dimensional diagrams of the substrates of maltotriose and maltose binding to α-amylase and α-glucosidase, respectively; (**C**,**D**) Three-dimensional and two-dimensional diagrams of flavonoids in BZYQF binding to α-amylase and α-glucosidase; Robinin and Sudachiin C are shown here; (**E**,**F**) The proportions of amino acid residues (interaction energy < −40, accounting for >5% of amino acid residues) in interactions, which formed between flavonoids of BZYQF and α-amylase or α-glucosidase. Among all flavonoids of BZYQF, Robinin and Sudachiin C had the lowest interaction energy to α-amylase (−73.7461) and α-glycosidase (−68.5875), respectively.

As shown in Figure 9A,C,E, similarly to the themaltotriose substrate, flavonoids of BZYQF (Robinin was shown) docked into the active site of α-amylase, exhibiting competitive binding behavior. When bound to α-amylase (interaction energy < −40), flavonoids of BZYQF were mainly surrounded by 13 amino acid residues of Glu 233, Lys 200, Asp 197, Asp 300, Tyr 151, Gln 63, His 201, Ala 198, Leu 162, Ile 235, Trp 62, Trp 59, and His 305. In these cases, five main interactions—Pi–anion, Pi–Pi (including stacked and T-shape), Pi–alkyl, hydrogen bonds, and carbon hydrogen bonds—were formed between flavonoids of BZYQF and α-amylase. In the proportion of amino acid residues in these five interactions, the proportions of Glu233, Asp197, Asp300, and His201 were higher than those of other amino acid residues, exceeding 50%. Moreover, the first three amino acid residues formed three interactions—Pi–anion, hydrogen bonds, and carbon hydrogen bonds—while the amino acid residue of His201 formed two interactions—Pi–Pi and carbon hydrogen bonds—between flavonoids of BZYQF and α-amylase. On binding to α-amylase, flavonoids of BZYQF hampered the access of the substrate or occupied the binding site of starch, which further suppressed the activity of α-amylase.

As shown in Figure 9B,D,F, unlike the substrate of maltose, flavonoids of BZYQF (Sudachiin C was shown) interacted with the essential amino acid residues located in the non-catalytic site of α-glucosidase, exhibiting a non-competitive or uncompetitive binding behavior. When bound to α-glycosidase (interaction energy < −40), flavonoids of BZYQF were mainly surrounded by 15 amino acid residues: Glu 856, Ile 823, Arg 725, Lyd 348, Glu 721, Arg 854, Glu 748, Gly 855, Gly 747, Pro 825, Leu 712, Ala 749, Tyr 822, His 708, and Ala 719. In these cases, five main interactions—Pi–anion, Pi–Pi, Pi–alkyl, hydrogen bonds, and carbon hydrogen bonds—were also formed between flavonoids of BZYQF and α-glycosidase. In the proportion of amino acid residues in these five interactions, the proportions of Glu748, Glu856, Tyr 822, and His 708 were higher than those of other amino acid residues, exceeding 50%. Moreover, the amino acid residues of Glu748 and Glu856 formed three interactions—Pi–anion, hydrogen bonds, and carbon hydrogen bonds—while the amino acid residues of Tyr 822 and His 708 formed two interactions—Pi–Pi and Pi–alkyl—between flavonoids of BZYQF and α-glycosidase. These amino acid residues increased the stability of the inhibitor–enzyme complex, exposing the inhibitory ability of flavonoids of BZYQF. On binding to α-glycosidase, flavonoids of BZYQF probably perturbed the protein structure and inhibited α-glucosidase in a non-competitive or uncompetitive mode, thus altering the enzyme activity.

## 3. Discussion

The present study found that BZYQF could significantly reduce postprandial hyperglycemia after the ingestion of starch solution in T2DM rats. The molecular mechanism may involve BZYQF inhibiting the activities of α-amylase and α-glycosidase, reducing glucose production and thereby preventing a sudden increase in blood glucose levels. Through multiple linear regression analysis and molecular docking, we speculated that the main constituents of BZYQF that inhibited α-amylase and α-glycosidase activities were total flavonoids. Moreover, this speculation was verified by analyzing the total flavonoids isolated from BZYQF. This study provides a theoretical basis for BZYQF to control postprandial hyperglycemia in T2DM.

Postprandial hyperglycemia is one of the central symptoms of T2DM. Controlling postprandial hyperglycemia can not only minimize blood glucose fluctuation but also reduce the risk of cardiovascular disease. However, the changes in postprandial blood glucose levels are closely associated with the composition of food and the efficiency of the body’s carbohydrate metabolism. Monosaccharide foods can be absorbed directly by the intestines, causing a rapid increase in blood glucose levels. Polysaccharide-rich foods such as starch must be enzymatically digested in the mouth and intestines to be converted into monosaccharides before absorption, resulting in a more consistent and gradual increase in blood glucose levels compared to monosaccharide foods. In addition, glucose was absorbed into the bloodstream via the SGLT1 and GLUT2 transporters in the small intestine, resulting in an increase in postprandial blood glucose [12]. The increased blood glucose stimulated pancreatic β-cells to secrete insulin, which, in turn, activated peripheral tissues to uptake glucose, thus lowering postprandial blood glucose to maintain glucose homeostasis. Therefore, the changes in postprandial blood glucose levels were influenced by food, digestive enzyme activity, glucose transporters, and the function of insulin. At present, in the clinical treatment of T2DM, most drugs target insulin and glucose transporters, such as metformin, thiazolidinediones, GLP-1 receptor agonists, SGLT-1 inhibitors, etc., while few drugs target digestive enzymes. Acarbose, as an α-glycosidase inhibitor, had a prominent effect on controlling postprandial hyperglycemia. However, its side effects, such as gastrointestinal complaints, allergic reactions, and damage to liver function, were also evident. Therefore, studying the glycemic capacity of carbohydrate-containing foods and screening for digestive enzyme inhibitors with low side effects remain important for the control of postprandial hyperglycemia in T2DM. BZYQF has been used as a classic formula of TCM in China for hundreds of years. It has been widely used to regulate subhealth and impaired spleen and stomach functions [13] and has excellent therapeutic effects in improving digestion and immune deficiency [14,15,16]. Clinical studies have found that BZYQF could improve fasting blood glucose and pancreatic islet function in patients with T2DM [17]. However, research on the role of BZYQF in regulating postprandial hyperglycemia in T2DM is limited. The present study found that following the ingestion of glucose and starch solutions, the blood glucose values of CON rats increased significantly from 5 mM to 7 mM, and those of T2DM rats increased from 20 mM to 25 mM. This result is supported by the reports of Momtazi-Borojen et al. [18] and Liu et al. [19], in which the blood glucose value of CON or T2DM rats had a similar increase from the low value to the highest value in an oral glucose tolerance test. These results indicate that the concentrations of glucose/starch solutions used in this study were effective. Furthermore, this study found that the blood glucose level and AUC of T2DM rats were significantly higher than those of CON rats. This result is also confirmed by a large number of animal experiments [20,21,22,23]. Interestingly, the blood glucose levels of CON rats had no difference between the ingestion of glucose and starch solutions, while T2DM rats had lower blood glucose levels following the ingestion of starch solution than those following the ingestion of glucose solution. These results suggest that CON rats were able to rapidly digest starch and utilize glucose, whereas T2DM rats exhibited abnormal carbohydrate metabolism, manifested by a reduced ability to digest starch and utilize glucose. As is well known, T2DM rats have insufficient insulin secretion and insulin resistance, and there are increased expressions of GLUT2 and SGLT-1 in the intestine of T2DM rats [24,25,26]. In addition, this study also found that after BZYQF intervention, BZYQF could significantly reduce the blood glucose levels at 15 min after the ingestion of starch solution and within 0–60 min after the ingestion of glucose solution in CON rats. However, BZYQF could only reduce the blood glucose levels at 15 min after the ingestion of starch solution and had no effect on the blood glucose levels within 0–45 min after the ingestion of glucose solution in T2DM rats. These results suggest that BZYQF could better stabilize postprandial blood glucose levels in CON rats and significantly reduce postprandial blood glucose following the ingestion of starch solution in CON and T2DM rats. BZYQF could reduce postprandial blood glucose levels following the ingestion of glucose solution in CON rats but not in T2DM rats. This might be because BZYQF regulated glucose uptake and improved insulin secretion and sensitivity in CON rats, while it did not improve impaired glucose utilization and insulin function in T2DM rats in the short term. Clinical observations have shown that BZYQF could improve insulin sensitivity and reduce hyperglycemia in T2DM patients [17]. Moreover, our previous research on *Astragalus membranaceus* (the most important Chinese herbs in BZYQF) found that after treatment for 8 weeks, Astragalus polysaccharides could promote insulin secretion, improve insulin resistance, and reduce the expressions of SGLT-1 and GLUT2 in the intestine of T2DM rats [27]. Notably, BZYQF could significantly reduce blood glucose levels at 60 min after the ingestion of glucose solution in T2DM rats. This result indicates that prolonging the intervention time of BZYQF had a certain improvement effect on glucose utilization in T2DM rats. In the present study, BZYQF simultaneously reduced the blood glucose levels at 15 min after the ingestion of starch solution in CON and T2DM rats, with a more significant decrease in blood glucose levels in T2DM rats. This result suggests that BZYQF could inhibit the activity of digestive enzymes and that T2DM rats exhibit defects in starch digestion. In addition, after intervention with BZYQF, the peak of blood glucose levels in CON and T2DM rats was delayed from 15 min to 30 min after the ingestion of starch solution, while similar results were not observed in rats that ingested glucose solution. This further confirms that BZYQF could inhibit the process of starch digestion into glucose. Clinical studies have found that ZBYQF reduces postprandial glucose levels within 2 h [9]. Thus, it can be seen that BZYQF could significantly reduce postprandial hyperglycemia in T2DM rats, and its mechanism might be related to the improvement of glucose metabolism, especially the inhibition of digestive enzyme activity.

Digestive enzymes, particularly α-amylase and α-glucosidase, are key enzymes involved in the hydrolysis of dietary starch to produce glucose, which has been closely linked to elevated postprandial blood glucose [28]. α-amylase catalyzes the hydrolysis of α-1,4-glycosidic bonds of starch, glycogen, and various oligosaccharides. α-glucosidase catalyzes the breaking of glycosidic bonds and the subsequent release of glucose from the non-reducing ends of oligosaccharide chains [28,29]. When dietary starch is ingested, it is first hydrolyzed by salivary α-amylase in the mouth and pancreatic α-amylase in the small intestine, yielding smaller oligomers such as maltose and maltotriose. Subsequently, these oligomers are further degraded into glucose molecules by other hydrolyzing enzymes, such as α-glucosidase, in the small intestine. Finally, glucose is transported into the bloodstream via the transporters of GLUT2 and SGLT1. Therefore, the inhibition of these enzyme activities could reduce postprandial blood glucose levels by delaying the digestion of carbohydrates into glucose. The IC50 value refers to the semi-inhibitory concentration of the measured antagonist. It represents the concentration at which a drug inhibits enzyme activity by 50%. In the drug screening process, IC50 values are used to preliminarily screen potential drug candidate molecules and determine their activity against specific targets. Usually, a smaller IC50 value indicates that the drug has a stronger inhibitory effect. Enzyme kinetics studies have shown that inhibition consists of both reversible and irreversible inhibition. Between the two, reversible inhibition is more important as it only slows down the digestive process but does not permanently block it. By slowing down the digestive process, glucose would be produced at a more consistent rate, thereby preventing blood sugar spikes [30]. Reversible inhibition includes three types: competitive, non-competitive, and uncompetitive. In competitive inhibition, the inhibitor and substrate have similar structures and compete for binding to the active sites of the enzyme. In non-competitive inhibition, both the substrate and the inhibitor bind to the enzyme at completely independent sites. In contrast, uncompetitive inhibitors do not bind to the free enzyme, but only to the enzyme–substrate complex [31,32]. Acarbose, which has been widely used in the clinical treatment of diabetes, is a classic inhibitor, binding competitively to α-amylase and α-glucosidase. The present study found that BZYQF had a good inhibitory effect on the activities of α-amylase and α-glucosidase in vitro, although the IC50 values of BZYQF against the two enzymes were higher than those of acarbose. Similarly to acarbose, BZYQF showed a stronger inhibitory effect on α-glucosidase than on α-amylase. In contrast to acarbose, BZYQF bound to α-amylase in a mixed competition mode, while it bound to α-glucosidase in an uncompetitive mode. Some studies have reported that a single constituent in BZYQF had inhibitory effects on α-amylase and α-glucosidase, although the inhibitory effects of BZYQF for the two enzymes have not been reported yet. Our previous study showed that *Astragalus* polysaccharides exerted an inhibitory effect on α-amylase in a dose-dependent manner [33]. Other research has reported that *Astragalus* polysaccharides and isoflavones have an inhibitory effect on α-glucosidase [34,35]. Licorice extracts have shown important anti-amylase and anti-glucosidase activities [36,37], among which glycyrrhizic acid inhibited α-glucosidase activity in a mixed competition mode [38]. *Codonopsis pilosula* (Franch.) Nannf. has been shown to have a strong inhibitory effect against mammalian α-glucosidase, which could significantly reduce the postprandial blood glucose level in diabetic mice in an oral starch tolerance test [39]. Therefore, BZYQF could lower postprandial blood glucose by inhibiting the activities of α-amylase and α-glucosidase, but the active constituents that inhibited enzyme activity need to be further determined.

Widely targeted metabolomic analysis and molecular docking have been widely used to identify active constituents in herbs and formulas. The results of our metabolomics analysis show that flavonoids accounted for the highest proportion among the 1909 compounds identified in BZYQF, accounting for over 40%, although their contents were not abundant. Using UPLC-QE-MS analysis, Xin Su et al. [40] also found that the chemical constituents of BZYQF included large amounts of flavonoids. In vitro simulated digestion assays simulated the physiological conditions of digestion in vivo and have been widely used in pharmacology to study and understand the changes, interactions, and bioavailability of chemical compounds. The present study found that the main constituent contents of BZYQF, excluding polysaccharides, did not significantly change during digestion with SSF, suggesting that SSF has a weak effect on BZYQF. The polysaccharide content decreased during salivary digestion, indicating that some polysaccharides could be hydrolyzed by amylase. During digestion with SGF, the total carbohydrate and reducing sugar contents in BZYQF increased, while polysaccharide contents decreased, indicating that glycans and polysaccharides were broken down into monosaccharides or oligosaccharides in SGF. Jing Kun Yan et al. [41] found that after digestion with SGF, the reducing sugar contents increased significantly, which might be due to the strong acidic environment in SGF disrupting glycosidic bonds, leading to an increase in reducing sugars. In this study, during digestion with SIF, the total carbohydrate and reducing sugar contents in BZYQF decreased. This could be because the small intestinal fluid affected the conformation and stability of polysaccharides, thereby leading to a reduction in reducing sugar content [42]. This study also found that the total flavonoid content of BZYQF significantly decreased after digestion with SGF, while there was no change after digestion with SIF. This result suggests that flavonoids in BZYQF could be degraded by the strong acids in SGF. A similar result was reported by Zhang et al. [43], where the total flavonoid content in the ethanol extract from camphor seed kernels decreased after gastric digestion. In addition, the present study found that the total polyphenol content in BZYQF increased during digestion with SIF, and the total saponin content in BZYQF had no significant change during salivary–gastrointestinal digestion. This result suggests that the total polyphenols in BZYQF could be influenced by SIF, while the total saponins in BZYQF were not affected by simulated digestive fluid. The increase in total polyphenol content could be due to the degradation of macromolecular polyphenols, releasing simpler polyphenolic acids. These polyphenol derivatives release active functional groups and react with Folin–Ciocalteu reagents to increase the total polyphenol content [44]. In vitro enzyme inhibition assays revealed that the inhibition rates of BZYQF against α-amylase and α-glucosidase varied with the changes in its main constituent contents during simulated digestion. Notably, the trend in changes in the BZYQF inhibition rates of α-amylase and α-glucosidase was consistent with the trend in changes in its total flavonoid content. Furthermore, correlation analysis and multiple linear regression analysis showed that the total flavonoid content in BZYQF was significantly positively correlated with its inhibition rates against α-amylase and α-glycosidase. These results indicate that the total flavonoids were the active constituents of BZYQF in inhibiting α-amylase and α-glycosidase activities. Subsequently, we extracted and isolated the total flavonoids from BZYQF and found that the IC50 values of the purified total flavonoids against α-amylase and α-glucosidase activities were significantly lower than those of BZYQF. This result indicates that the purified total flavonoids had stronger enzyme inhibitory activities than BZYQF, which further verified the inhibitory effects of total flavonoids in BZYQF on the activities of α-amylase and α-glucosidase. Finally, our molecular docking results indicate that 40% of the flavonoids in BZYQF could bind to α-amylase and α-glycosidase simultaneously and that these flavonoids bound more strongly to α-glycosidase than to α-amylase. This result is consistent with the lower IC50 values of BZYQF and the purified total flavonoids against α-glycosidase than their IC50 values against α-amylase. In addition, the molecular docking site of this study showed that flavonoids in BZYQF were bound to the inactive site of α-glucosidase while they were bound to the active site of α-amylase. This result is consistent with the results of the enzyme kinetics assay, in which BZYQF inhibited α-glucosidase activity in an uncompetitive mode, and α-amylase activity, in a mixed competition and non-competition mode. At the same time, this result also suggests that among the main constituents in BZYQF that inhibited α-amylase activity, in addition to flavonoids, other chemical constituents such as polysaccharides and polyphenols could exert their effects in a non-competitive mode. Our previous study found that *Astragalus* polysaccharides had an inhibitory effect on α-amylase activity [33]. A review confirmed that polyphenols from various extracts, such as gallic acid, caffeic acid, and kaempferol, inhibited α-amylase activity in a non-competitive mode [45]. In addition, several studies have shown that flavonoids have a strong inhibitory effect on α-glucosidase and a moderate inhibitory effect on α-amylase, making them promising candidates for antidiabetic drugs [46,47,48]. However, no consensus has been reached regarding the mode by which flavonoids inhibit α-glucosidase and α-amylase. This could be due to the differences in plant flavonoid sources and detection methods used in different studies [46]. Overall, flavonoids play an important role in the inhibition of α-glucosidase and α-amylase activity in BZYQF.

## 4. Materials and Methods

### 4.1. Preparation of BZYQF

BZYQF was purchased from JiuZhiTang Co., Ltd. (National Medical Approval No. Z43020143, Changsha, China). It was composed of 8 Chinese herbs, including 200 g of *Astragalus membranaceus* (Fisch.) Bunge (honey roasted), 100 g of *Glycyrhiza uralensis* Fisch. (honey roasted), 60 g of *Codonopsis pilosula* (Franch.) Nannf., 60 g of *Atractylodes macrocephala* Koidz. (stir fried), 60 g of *Angelica sinensis [Oliv.] Diels*., 60 g of *Cimicifuga heracleifolia* Kom, 60 g of *Bupleurum chinense* DC., 60 g of *Citrus reticulata* Blanco., 40 g of *Ziziphus jujuba* Mill, and 20 g of *Zingiber offcinale* Roscoe. BZYQF was manufactured in accordance with the drug standards of the Ministry of Health for Traditional Chinese Medicine Formulations (Volume 7). A brief introduction is as follows: the volatile oil was extracted from *Zingiber officinale* and *Citrus reticulata*. The remaining residues were boiled twice in water with *Astragalus membranaceus*, *Atractylodes macrocephala*, *Cimicifuga heracleifolia*, *Bupleurum chinense*, *Ziziphus jujuba*, and one half of *Glycyrhiza uralensis*, for 3 h and 2 h, respectively. The decoction was collected and filtered. The filtrate was concentrated into a thick paste with a relative density of 1.3–1.35. The *Codonopsis pilosula*, *Angelica sinensis*, and the other half of *Glycyrhiza uralensis* were crushed into fine powder. All the above pastes, powders, and volatile oils were mixed to make BZYQF pills.

### 4.2. Characterization of Chemical Constituents in BZYQF

A chemical constituent analysis of BZYQF was conducted using the widely targeted metabolomic techniques reported by Chen et al. [49]. A brief introduction is as follows: BZYQF was ground into powder (30 Hz, 1.5 min) using a grinder (MM 400, Retsch, Haan, Germany) after being freeze-dried in a lyophilizer (Scientz-100F, Ningbo, China). A measure of 50 mg of sample powder was added to 1200 μL of pre-cooled 70% methanolic aqueous solution and vortexed for 30 s once every 30 min a total of 6 times. After centrifugation at 12,000× *g* for 3 min, the supernatant was filtered with a microporous membrane (0.22 μm pore size) for UPLC-MS/MS analysis. The UPLC analytical conditions were as follows: the injection volume was 2 μL; the flow velocity was set as 0.35 mL/min; the column oven was set at 40 °C; an Agilent SB-C18 column (1.8 µm, 2.1 mm × 100 mm, Agilent, Santa Clara, CA, USA) was used; and the mobile phase consisted of solvent A, pure water with 0.1% formic acid, and solvent B, acetonitrile with 0.1% formic acid. Sample measurements were carried out using a gradient program as follows: the initial conditions of 95% A and 5% B were used. A linear gradient to 5% A and 95% B was then programmed within 9 min, and a composition of 5% A and 95% B was maintained for 1 min. Subsequently, a composition of 95% A and 5.0% B was adjusted within 1.1 min and maintained for 2.9 min. The effluent was alternatively connected to an ESI-Triple Quadrupole-Linear Ion Trap (QTRAP)-MS (Qtrap 5500, AB Sciex, Framingham, MA, USA). The operating parameters of the ESI source were as follows: the source temperature was 500 °C; the ion spray voltage (IS) was 5500*V* (positive ion mode)/−4500*V* (negative ion mode); ion source gas I (GSI), gas II (GSII), and curtain gas (CUR) were set at 50, 60, and 25 psi, respectively; collision-activated dissociation (CAD) was set to high; QQQ scans were performed using the MRM model; and collision gas (nitrogen) was set to medium. The DP (declustering potential) and CE (collision energy) for individual MRM transitions were acquired through DP and CE optimization. A specific set of MRM ion pairs were monitored at each period according to the eluted metabolites during this period. The primary and secondary metabolites were determined using secondary spectral information and the self-created database MWDB (Metware database, Wuhan, China).

The content of calycosin-7-O-β-D-glucoside, an isoflavone isolated from *Astragalus membranaceus* in BZYQF, was determined using HPLC (UltiMate3000, Thermo, Waltham, MA, USA). After treatment with methanol, BZYQF and the standard samples of calycosin-7-O-β-D-glucoside were filtered using a 0.22 μm microporous filter membrane, and the filtrate was analyzed using HPLC. The HPLC analytical conditions were as follows: the injection volume was 20 μL; the flow velocity was set as 1 mL/min; the column oven was set at 30 °C; an Agilent Durashell C18(L) column (1.5 µm, 4.6 mm × 250 mm) was used; and the mobile phase was acetonitrile with 0.1% phosphoric acid solution (3:97). Sample measurements were performed using gradient elution with a wavelength range of 200–400 nm.

### 4.3. Quantitative Determination of the Main Constituents in BZYQF

BZYQF pills were crushed and then incubated in water at 90 °C at a solid/liquid ratio of 1:30 for 1 h. After centrifugation at 3000× *g* for 10 min, the supernatant was used for subsequent experiments, including the following assays for quantitation, enzyme inhibition, and simulated digestion.

The total carbohydrate content was determined using the phenol–sulfuric acid method [50]. BZYQF solutions or standard solutions (1 mL) were mixed with 0.5 mL of 6% phenol solution and 2.5 mL of concentrated sulfuric acid. The mixture was placed into boiling water for 15 min and quickly cooled to room temperature with cold water. The absorbance at 490 nm was measured on a microplate reader (ELx800, BIOTEK, Winooski, VT, USA). The total carbohydrate content was calculated from a standard glucose curve and expressed as a glucose equivalent (mg/g crude drug).

The reducing sugar content was determined using the 3,5-dinitrosalicylic acid method [50]. BZYQF solutions or standard solutions (2 mL) were mixed with 2 mL of 3,5-dinitrosalicylic acid. The mixture was placed into boiling water for 5 min and quickly cooled to room temperature. The total volume of the mixture was supplemented to 25 mL by adding 21 mL of distilled water. The absorbance at 520 nm was measured on a microplate reader (ELx800, BIOTEK, Vermont, USA). The reducing sugar content was calculated from a standard glucose curve and expressed as a glucose equivalent (mg/g crude drug).

The polysaccharide content was calculated as the difference between the total carbohydrate content and reducing sugar content.

The total flavonoid content was determined using a colorimetric method with minor modifications [51]. BZYQF solutions or standard solutions (1 mL) were mixed with 0.3 mL of 5% NaNO_2_ solution (*w*/*v*) and incubated for 6 min at room temperature. Then, 0.3 mL of 5% Al(NO_3_)_3_ solution (*w*/*v*) was added and incubated for 6 min at room temperature. Finally, 4 mL of 4% NaOH (*w*/*v*) was added and incubated for 15 min at room temperature. The absorbance at 510 nm was determined on a microplate reader (ELx800, BIOTEK, Vermont, USA). The total flavonoid content was calculated from a standard rutin curve and expressed as a rutin equivalent (mg/g crude drug).

The total polyphenol content was determined using Singleton’s method [52], with minor modifications. BZYQF solutions or standard solutions (1 mL) were mixed with 0.25 mL of 50% Folin–Ciocalteu reagent and 0.75 mL of 20% sodium carbonate solution. The mixture was kept in the dark at room temperature for 30 min. The absorbance at 740 nm was measured on the microplate reader (ELx800, BIOTEK, Vermont, USA). The total polyphenol content was calculated from a standard gallic acid curve and expressed as a gallic acid equivalent (mg/g crude drug).

The total saponin content was determined using Milad Hadidi et al.’s method [53], with minor modifications. BZYQF solutions or standard solutions (1 mL) were mixed with 0.5 mL of 8% vanillin ethanol solution (*w*/*v*) and 5 mL of 72% sulfuric acid. The mixture was heated for 10 min at 60 °C, followed by cooling on ice for 5 min. The absorbance at 520 nm was measured on a microplate reader (ELx800, BIOTEK, Vermont, USA). The total saponin content was calculated from a standard ginsenoside curve and expressed as a ginsenoside equivalent (mg/g crude drug).

### 4.4. Induction of T2DM Rats

Male Sprague Dawley (SD) rats (190–210 g) were provided by the Medical Experimental Animal Center of Guangdong Province, with production license number SCXK (Guangdong) 2022-0002. SD rats were bred in the Experimental Animal Center of Guangdong Pharmaceutical University at a relative humidity of 65% ± 5% and 20 ± 2 °C room temperature with a 12/12 h light/dark cycle. The present study strictly complied with animal welfare ethics about the reasonable use of animal numbers and measures to reduce animal suffering, and it was approved by the Laboratory Animal Ethics Committee of Guangdong Pharmaceutical University (approval number gdpulacspf2022203). T2DM induction was performed according to references for previously validated models [54,55]. After adaptive feeding for 3 days, SD rats were randomly divided into two groups: control (CON, *n* = 8) and T2DM (*n* = 8). During the experiment, the rats in the CON group were fed a basal diet (25% flour, 25% cornmeal, 25% wheat, 10% bean power, 8% fish flour, 4% bone meal, 2% yeast, and 1% refined salt), while the rats in the T2DM group were fed a high-sugar and high-fat diet (20% casein, 17.8% lard, 16.8% sucrose, 10% maltodextrin, 7.3% corn starch, 5% cellulose, 5% mineral mix S10026B, 2.5% soybean oil, 1% vitamin mix ain-76A, 0.3% L-cystine, and 0.2% choline bitartrate). After feeding for 8 weeks, the rats in the T2DM group received a single intraperitoneal injection of 35 mg/kg streptozotocin (STZ, Sigma, Saint Louis, MO, USA), and the rats in the CON group were injected with an equivalent volume of citrate buffer after fasting for 12 h. After STZ injection for one week, orbital blood was collected to measure fasting blood glucose (FBG). The T2DM rat model was successfully induced when rats had FBG ≥ 11.1 mmol/L and lower fasting blood insulin and HOMA-IS than CON rats, accompanied by typical symptoms such as polydipsia, polyphagia, polyuria, and weight loss. After completing the experiment on postprandial glycemic responses following carbohydrate ingestion, experimental rats were anesthetized through the intraperitoneal injection of 10% pelltobarbitalum natricum. After anesthesia was determined through the pain reflex and cornea reflex, experimental rats were sacrificed by draining blood from the abdominal aorta.

### 4.5. Postprandial Glycemic Responses Following Carbohydrate Ingestion

In vivo postprandial glycemic responses following the ingestion of starch and glucose solutions were recorded according to our previous method [33] and Mandel AL and Breslin PA’s method [56], with minor modifications. The four independent experiments were conducted in the mornings on separate days. After fasting for 12 h, each rat was administered 0.5 g/kg (7.5% solution) of glucose/starch solution in the control condition, and 0.5 g/kg of glucose/starch solution + 8 g/kg BZYQF in the experimental condition through a single oral dose. The blood glucose value was measured in serum samples from the tail vein at 0, 15, 30, 45, and 60 min. The area under the curve (AUC) was calculated using the trapezoid method. The glucose and starch solutions were equal in terms of energy provided. Before use, soluble starch was dissolved in a small amount of boiling water and then prepared to a concentration of 7.5%. This starch was processed to obtain a solution that was not noticeably different in viscosity from the glucose solution. A BZYQF dosage of 8 g/kg was selected according to the reference confirmed by our preliminary experimental results [57]. One hour after a meal is an important stage for food digestion and absorption, during which blood glucose rapidly increases and reaches its peak, reflecting individual blood glucose fluctuations. Hence, the present study mainly observed the 1 h postprandial blood glucose change.

### 4.6. In Vitro Inhibition Assays of α-Amylase and α-Glucosidase

The inhibition assay of α-amylase and α-glucosidase was carried out according to the method described by Yuxue Zheng et al. [58] with some modification. Briefly, 50 μL measures of α-amylase (meilunbio Co., Ltd., Dalian, China, 10 U/mL, dissolved in phosphate-buffered saline with pH 6.9) were mixed thoroughly with 50 μL measures of BZYQF solutions (0, 1, 10, 30, 40, 50, 70, 77 mg/mL, dissolved in double distilled water) and incubated for 10 min at 37 °C. Then, 0.1 mL of soluble starch (Macklin Biochemical Technology Co., Ltd., Shanghai, China, 1%, dissolved in double-distilled water) was added and incubated for 10 min at 37 °C. Finally, 0.1 mL of DNS reagent (3,5-dinitrosalicylic acid, Phygene Biotechnology Co., Ltd., Fuzhou, China) was added and incubated for 5 min at 100 °C for color development. The absorbance of the colored complex was recorded at 540 nm using an Elx-800 microplate reader (BioTek, Vermont, USA).

The inhibitory assay of α-glucosidase was performed as follows. Briefly, 50 μL of α-glucosidase (Yuanye Bio-Technology Co., Ltd., Shanghai, China, 4.4 U/mL, dissolved in PBS with pH 6.9) was mixed thoroughly with 50 μL of BZYQF (0, 1, 4, 7, 10, 30, 40, 77 mg/mL, dissolved in double-distilled water) and incubated for 10 min at 37 °C. Then, 0.1 mL of pNPG solution (2.5 mmol/L) as the substrate was added and incubated for 10 min at 37 °C. Finally, the reaction was terminated by adding 0.1 mL of 0.2 mol/L Na_2_CO_3_. The absorbance of the solution was recorded at 450 nm using an Elx-800 microplate reader (BioTek, Vermont, USA).

The inhibition rate of BZYQF for α-amylase and α-glucosidase was calculated according to Equation (1):

(1) $$\mathrm{Inhibition}\%=\frac{\left(\mathrm{Acontrol}-\mathrm{Acontrol}\;\mathrm{blank}\right)-\left(\mathrm{Asample}-\mathrm{Asample}\;\mathrm{blank}\right)}{\mathrm{Acontrol}-\mathrm{Acontrol}\;\mathrm{blank}}$$

where A_control_, A_control blank_, A_sample_, and A_sample blank_ represent the absorbance values of the sample with enzymes and PBS, PBS without enzymes, BZYQF with enzymes, and BZYQF without enzymes, respectively. Acarbose was used as a positive control. All samples were measured in triplicate. The IC50 value is the concentration of BZYQF required for 50% inhibition of the α-amylase/α-glucosidase activity.

### 4.7. Kinetic Inhibition Mode of α-Amylase and α-Glucosidase

The inhibition mode of α-amylase and α-glucosidase by BZYQF was determined using the Lineweaver–Burk plot method at specific enzyme concentrations and reaction times [58]. The concentrations of the starch solution ranged from 2.5 to 10.0 mg/mL, while the concentrations of the pNPG solution ranged from 0.5 to 2.5 mmol/L, and the crude drug concentrations of BZYQF solutions ranged from 0 to 50 mg/mL. The assay was performed as mentioned in Section 4.6. The Michaelis constant (Km) and maximal velocity (Vmax) were obtained from the plots.

### 4.8. In Vitro Simulated Digestion of BZYQF

The in vitro simulated digestion procedure was carried out according to the INFOGEST protocol with some modifications [59]. Simulated salivary fluid (SSF), simulated gastric fluid (SGF), and simulated intestinal fluid (SIF) were purchased from Yuanye Bio-Technology Co., Ltd. (Shanghai, China). A measure of 8 mL of SSF, including KCl, KSCN, NaH_2_PO_4_, Na_2_SO_4_, NaCl, NaHCO_3_, amylase, uric acid, and mucin, was preheated in water for 5 min at 37 °C and added to 16 mL of BZYQF solution (as mentioned in Section 4.3). The mixture was incubated for 5 min at 37 °C. After the incubation, 8.0 mL of the digested sample was taken out and immediately placed in boiling water for 5 min to inactivate the enzymes. The remaining 16 mL of digestion mixture was retained to simulate gastric digestion.

Hydrochloric acid of 6 mol/L was added to the digestion mixture of saliva to bring the pH to 2.0. A measure of 8 mL of SGF, including NaCl, KCl, CaCl_2_, NH_4_Cl, NaH_2_PO_4_, glucose, glucuronic acid, HCl, urea, BSA, pepsin, and mucin, was preheated in water for 5 min at 37 °C and added to the digestion mixture of saliva with pH 2.0. Then, the mixture was incubated for 2 h at 37 °C. After the incubation, 8 mL of the digested sample was taken out and immediately placed in boiling water for 5 min to kill the enzymes. The remaining 16 mL of digestion mixture was retained to simulate intestinal digestion.

The pH in the gastric digestion mixture was adjusted to 7.0 with 6 mol/L sodium hydroxide. A measure of 8 mL of SIF, including NaCl, KCl, CaCl_2_, NaOH, NaHCO_3_, phosphate, and pancreatin, was preheated in water for 5 min at 37 °C and added to the gastric digestion mixture with pH 7.0. Then, the mixture was incubated for 2 h at 37 °C. After the incubation, 8 mL of the digested sample was taken out and immediately placed in boiling water for 5 min to inactivate the enzymes.

During the in vitro simulated digestion, ultrapure water was added to the simulated digestive fluids as a no-BZYQF control. BZYQF solution was mixed with ultrapure water as a control without simulated digestive fluids. All experiments were repeated six times.

### 4.9. Extraction and Isolation of Total Flavonoids and In Vitro Activity Validation

The extraction and isolation of total flavonoids were carried out according to the method reported by Yao et al. [60], with some modifications. The BZYQF pills were crushed to a powder and were extracted exhaustively with 50% aqueous ethanol (with a liquid-to-material ratio of 30:1, *v*/*w*) at 50 °C for 1 h through reflux extraction on three occasions. After centrifugation at 3000× *g* for 10 min, the supernatant was concentrated under reduced pressure in a rotary evaporator at 40 °C, and the crude extracts were obtained. These crude extracts were then chromatographed on a glass column (60 cm × 3 cm, containing 150 g of D101 macroporous resin) with a 0.2 L bed volume (BV) for 7 h of adsorption. After reaching adsorption equilibrium, distilled water was used to wash the D101 macroporous resin until the effluent was almost colorless. Finally, 3.5 BV of 50% aqueous ethanol was used to rinse the adsorbent with an elution rate of 1 mL/min. The elution of 50% aqueous ethanol was collected in separate test tubes with 30 mL/tube, and then concentrated and dried. The purified total flavonoid was used to determine the main constituent content and perform in vitro inhibition assays of α-amylase and α-glucosidase as described in Section 4.3 and Section 4.6.

### 4.10. Molecular Docking

Molecular docking was applied to speculate the possible binding site(s) between flavonoids of BZYQF and α-amylase/α-glucosidase. The crystal structures of α-amylase (PDB code: 5E0F) and α-glucosidase (PDB code: 5NN8) were downloaded from the RCSB Protein Data Bank “https://www.rcsb.org/ (accessed on 2 May 2024)”. The 2D structures of flavonoids in BZYQF were downloaded from the PubChem “https://pubchem.ncbi.nlm.nih.gov/ (accessed on 10 May 2024)”, TCMSP “https://www.tcmsp-e.com/#/home (accessed on 10 May 2024)” and ZINC “https://zinc20.docking.org/ (accessed on 10 May 2024)” databases. Autodock version 4.2.6 was used to remove the cofactors, water molecules, and heteroatoms from 5E0F and 5NN8 and obtain a stable receptor for flavonoids of BZYQF. The optimized protein structures of 5NN8 and 5E0F were imported into Discovery Studio 2019 “http://www.discoverystudio.net (accessed on 12 May 2024)” for batch docking with pre-processed flavonoids in BZYQF. The CDOCKER module of Discovery Studio 2019 was used to analyze the interaction energy of α-amylase and α-glucosidase with flavonoids in BZYQF. The parameters (e.g., binding sites of amino acid residues and interactions) were obtained based on the docking results with CDOCKER interaction energy of <−40 Kcal/mol.

### 4.11. Statistical Analysis

The comparison analysis of multiple groups was conducted using one-way ANOVA with Tukey’s post hoc test, while the comparison between two groups was conducted using Student’s *t*-test with GraphPad Prism version 8 (GraphPad Software, La Jolla, CA, USA). The active constituents in BZYQF inhibiting the activities of α-amylase and α-glucosidase were identified through Pearson correlation analysis and multiple linear regression analysis using Origin Pro 2021 (OriginLab, Northampton, MA, USA) and SPSS version 22.0 (IBM SPSS, New York, NY, USA), respectively. The 50% inhibitory concentration (IC50) of BZYQF against the activities of α-amylase and α-glucosidase was calculated through nonlinear regression using GraphPad Prism version 8. *p* < 0.05 was considered statistically significant. All data are presented as the mean ± standard deviation ($\bar{x}$ ± SD).

## 5. Conclusions

In summary, the present study demonstrated that BZYQF could significantly reduce postprandial hyperglycemia following the ingestion of starch solution in T2DM rats by inhibiting α-amylase and α-glycosidase activities. An in vitro enzyme kinetic assay suggested that BZYQF inhibited α-glucosidase activity in the uncompetitive mode and inhibited α-amylase activity in the competitive and non-competitive modes. Furthermore, BZYQF had a stronger inhibitory effect on α-glucosidase activity than on α-amylase activity. These results were also theoretically validated through molecular docking results. The Pearson correlation and multiple linear regression analysis results revealed that the total flavonoids in BZYQF were the active constituents inhibiting α-amylase and α-glycosidase activities. This result was verified by examining the total flavonoids purified from BZYQF. Overall, the present study provided a theoretical basis for the use of BZYQF as an adjuvant therapy in T2DM. In addition, the effect of the total flavonoids in BZYQF on postprandial hyperglycemia in T2DM rats needs further confirmation through in vivo experiments.

## Figures and Tables

**Table 1:** Kinetic parameters of α-amylase and α-glucosidase inhibitions by BZYQF.

Enzyme	BZYQF Con. (mg/mL)	Km (mg/mL)	Vmax (ΔOD/min)	Slope
α-amylase	0	9.16 ± 0.94	0.30 ± 0.02	30.53 ± 0.95
	10	8.08 ± 1.07	0.24 ± 0.02	33.52 ± 1.47
	30	7.13 ± 2.03	0.19 ± 0.04	36.94 ± 3.66
	50	6.18 ± 1.51	0.15 ± 0.02	40.81 ± 3.85
α-glucosidase	0	9.36 ± 1.39	1.18 ± 0.22	7.96 ± 0.12
	10	2.03 ± 0.22	0.27 ± 0.02	7.56 ± 0.29
	20	1.67 ± 0.25	0.21 ± 0.03	7.79 ± 0.48
	40	0.95 ± 0.15	0.12 ± 0.01	7.70 ± 0.69

**Table 2:** Summary output of the multiple linear regression analysis performed between the inputs (the main constituent contents of BZYQF) and the output (the inhibition rates of α-amylase or α-glucosidase).

Enzyme	Variables	Regression Coefficient	*p* Value	95% CI (Lower ~ Upper)
α-amylase	Intercept	0.319	0.000	0.194 ~ 0.445
	Total carbohydrate	-	-	-
	Reducing sugar	0.014	0.200	−0.008 ~ 0.036
	Polysaccharide	0.023	0.266	−0.019 ~ 0.065
	Total flavonoid	0.294	0.010	0.082 ~ 0.507
	Total polyphenol	−0.057	0.072	−0.120 ~ 0.006
	Total saponin	0.111	0.427	−0.177 ~ 0.400
α-glucosidase	Intercept	0.532	0.000	0.448 ~ 0.616
	Total carbohydrate	-	-	-
	Reducing sugar	−0.010	0.191	−0.024 ~ 0.005
	Polysaccharide	0.017	0.228	−0.011 ~ 0.045
	Total flavonoid	0.236	0.003	0.094 ~ 0.378
	Total polyphenol	−0.018	0.386	−0.060 ~ 0.024
	Total saponin	−0.131	0.168	−0.324 ~ 0.061

**Table 3:** Summary output of the multiple linear regression analysis performed between the inputs (the main constituent contents of the total flavonoids purified from BZYQF) and the output (the inhibition rates of α-amylase or α-glucosidase).

Enzyme	Variables	Regression Coefficient	*p* Value	95% CI (Lower ~ Upper)
α-amylase	Intercept	0.003	0.957	−0.099 ~ 0.104
	Total carbohydrate	-	-	-
	Reducing sugar	−0.025	0.492	−0.103 ~ 0.053
	Polysaccharide	0.081	0.201	−0.051 ~ 0.213
	Total flavonoid	0.046	0.001	0.024 ~ 0.068
	Total polyphenol	0.003	0.696	−0.012 ~ 0.018
	Total saponin	0.111	0.251	−0.092 ~ 0.313
α-glucosidase	Intercept	0.219	0.018	0.046 ~ 0.392
	Total carbohydrate	-	-	-
	Reducing sugar	0.122	0.135	−0.045 ~ 0.289
	Polysaccharide	−0.155	0.216	−0.415 ~ 0.106
	Total flavonoid	0.051	0.017	0.011 ~ 0.091
	Total polyphenol	−0.005	0.735	−0.037 ~ 0.027
	Total saponin	−0.217	0.285	−0.645 ~ 0.211

## Data Availability

The datasets used and/or analyzed during the current study are available from the corresponding author on reasonable request.

## Supplementary Materials

The following supporting information can be downloaded at https://www.mdpi.com/article/10.3390/ph18020201/s1: Figure S1: Content of calycosin-7-O-β-D-glucoside in BZYQF; Figure S2: Content of standard (calycosin-7-O-β-D-glucoside); Figure S3: MRM_detection_of_multimodal_maps-N; Figure S4: MRM_detection_of_multimodal_maps-P; Figure S5: MWXS-23-777-01-a_QC_MS_TIC-N; Figure S6: MWXS-23-777-01-a_QC_MS_TIC-P; Table S1: All compounds were identified in BZYQF by UPLC-MSMS.

## References

[1] ElSayed N.A., Aleppo G., Aroda V.R., Bannuru R.R., Brown F.M., Bruemmer D., Collins B.S., Hilliard M.E., Isaacs D., Johnson E.L. (2023). 2. Classification and Diagnosis of Diabetes: Standards of Care in Diabetes-2023. Diabetes Care.

[2] Eid S., Sas K.M., Abcouwer S.F., Feldman E.L., Gardner T.W., Pennathur S., Fort P.E. (2019). New insights into the mechanisms of diabetic complications: Role of lipids and lipid metabolism. Diabetologia.

[3] Bonora E., Muggeo M. (2001). Postprandial blood glucose as a risk factor for cardiovascular disease in Type II diabetes: The epidemiological evidence. Diabetologia.

[4] Ni Y., Wu X., Yao W., Zhang Y., Chen J., Ding X. (2024). Evidence of traditional Chinese medicine for treating type 2 diabetes mellitus: From molecular mechanisms to clinical efficacy. Pharm. Biol..

[5] Chen X., Chen C., Fu X. (2022). Hypoglycemic activity in vitro and vivo of a water-soluble polysaccharide from Astragalus membranaceus. Food Funct..

[6] Guo J., Tao H., Cao Y., Ho C.T., Jin S., Huang Q. (2016). Prevention of Obesity and Type 2 Diabetes with Aged Citrus Peel (Chenpi) Extract. J. Agric. Food Chem..

[7] Xu J., Liu H., Su G., Ding M., Wang W., Lu J., Bi X., Zhao Y. (2021). Purification of ginseng rare sapogenins 25-OH-PPT and its hypoglycemic, antiinflammatory and lipid-lowering mechanisms. J. Ginseng Res..

[8] Yang L., Yu H., Hou A., Man W., Wang S., Zhang J., Wang X., Zheng S., Jiang H., Kuang H. (2021). A Review of the Ethnopharmacology, Phytochemistry, Pharmacology, Application, Quality Control, Processing, Toxicology, and Pharmacokinetics of the Dried Rhizome of Atractylodes macrocephala. Front. Pharmacol..

[9] Lai L., Hu T., Peng L. (2021). Analysis of the Dialectical Addition and Subtraction of Buzhong Yiqi Decoction on the Level of Blood Glucose Control and the Improvement of TCM Syndrome Scores in Patients with Type 2 Diabetes. Diabetes New World.

[10] Tolmie M., Bester M.J., Apostolides Z. (2021). Inhibition of α-glucosidase and α-amylase by herbal compounds for the treatment of type 2 diabetes: A validation of in silico reverse docking with in vitro enzyme assays. J. Diabetes.

[11] Dirir A.M., Daou M., Yousef A.F., Yousef L.F. (2022). A review of alpha-glucosidase inhibitors from plants as potential candidates for the treatment of type-2 diabetes. Phytochem. Rev..

[12] Proença C., Ribeiro D., Freitas M., Fernandes E. (2022). Flavonoids as potential agents in the management of type 2 diabetes through the modulation of α-amylase and α-glucosidase activity: A review. Crit. Rev. Food Sci. Nutr..

[13] Zheng X.F., Tian J.S., Liu P., Xing J., Qin X.M. (2014). Analysis of the Restorative Effect of Bu-Zhong-Yi-Qi-Tang in the Spleen-Qi Deficiency Rat Model Using 1H-NMR-Based Metabonomics. J. Ethnopharmacol..

[14] Hu L.F., Yamamoto M., Chen J.L., Duan H.F., Du J., He L.L., Shi D.F., Yao X.S., Nagai T., Kiyohara H. (2022). Integrating network pharmacology and experimental verification to decipher the immunomodulatory effect of Bu-Zhong-Yi-Qi-Tang against poly (I:C)-induced pulmonary inflammation. Front. Pharmacol..

[15] Mayu I., Yosuke T., Haruna H., Kayako S., Satoshi I., Yuki N., Michiko S., Takayuki N., Hiroaki K. (2020). Inulooligosaccharides in Atractylodis lanceae rhizoma and Atractylodis rhizoma of hochuekkito formula are essential for regulation of the pulmonary immune system of immuno-compromised mice. Tradit. Kampo Med..

[16] Zhang Y.G., Niu J.T., Zhang S.J., Si X.L., Bian T.T., Wu H.W., Li D.H., Sun Y.J., Jia J., Xin E.D. (2022). Comparative study on the gastrointestinal- and immune- regulation functions of Hedysari Radix Paeparata Cum Melle and Astragali Radix Praeparata cum Melle in rats with spleen-qi deficiency, based on fuzzy matter-element analysis. Pharm. Biol..

[17] Li N., Duan C., Hu M. (2019). Effect of Buzhong Yiqi decoction on blood glucose and islet function in patients with type 2 diabetes. Shanxi J. Tradit. Chin. Med..

[18] Momtazi-Borojeni A.A., Jaafari M.R., Abdollahi E., Banach M., Sahebkar A. (2021). Impact of PCSK9 Immunization on Glycemic Indices in Diabetic Rats. J. Diabetes Res..

[19] Liu K.-F., Niu C.-S., Tsai J.-C., Yang C.-L., Peng W.-H., Niu H.-S. (2022). Comparison of area under the curve in various models of diabetic rats receiving chronic medication. Arch. Med. Sci..

[20] Chewchinda S., Leakaya N., Sato H., Sato V.H. (2021). Antidiabetic effects of (Lour.) corner heartwood extract. J. Tradit. Compl. Med..

[21] Io F., Gunji E., Koretsune H., Kato K., Sugisaki-Kitano M., Okumura-Kitajima L., Kimura K., Uchida S., Yamamoto K. (2019). SGL5213, a novel and potent intestinal SGLT1 inhibitor, suppresses intestinal glucose absorption and enhances plasma GLP-1 and GLP-2 secretion in rats. Eur. J. Pharmacol..

[22] Ramkumar S., Thulasiram H.V., RaviKumar A. (2021). Improvement in serum amylase and glucose levels in diabetic rats on oral administration of bisdemethoxycurcumin from *Curcuma longa* and limonoids from *Azadirachta indica*. J. Food Biochem..

[23] Zhao X., Tao J., Zhang T., Jiang S., Wei W., Han H., Shao Y., Zhou G., Yue H. (2019). Resveratroloside Alleviates Postprandial Hyperglycemia in Diabetic Mice by Competitively Inhibiting α-Glucosidase. J. Agric. Food Chem..

[24] Burant C.F., Flink S., DePaoli A.M., Chen J., Lee W.S., Hediger M.A., Buse J.B., Chang E.B. (1994). Small intestine hexose transport in experimental diabetes. Increased transporter mRNA and protein expression in enterocytes. J. Clin. Investig..

[25] Dyer J., Wood I.S., Palejwala A., Ellis A., Shirazi-Beechey S.P. (2002). Expression of monosaccharide transporters in intestine of diabetic humans. Am. J. Physiol. Gastrointest. Liver Physiol..

[26] Fiorentino T.V., Suraci E., Arcidiacono G.P., Cimellaro A., Mignogna C., Presta I., Andreozzi F., Hribal M.L., Perticone F., Donato G. (2017). Duodenal Sodium/Glucose Cotransporter 1 Expression Under Fasting Conditions Is Associated With Postload Hyperglycemia. J. Clin. Endocrinol. Metab..

[27] Ze-Min Y., Ying W., Siyu C. (2021). Astragalus polysaccharide alleviates type 2 diabetic rats by reversing the glucose transporters and sweet taste receptors/GLP-1/GLP-1 receptor signaling pathways in the intestine-pancreatic axis. J. Funct. Foods.

[28] Shori A.B. (2015). Screening of antidiabetic and antioxidant activities of medicinal plants. J. Integr. Med..

[29] Borges de Melo E., da Silveira Gomes A., Carvalho I. (2006). α- and β-Glucosidase inhibitors: Chemical structure and biological activity. Tetrahedron.

[30] Campbell L.K., White J.R., Campbell R.K. (1996). Acarbose: Its role in the treatment of diabetes mellitus. Ann. Pharmacother..

[31] Masson P., Mukhametgalieva A.R. (2023). Partial Reversible Inhibition of Enzymes and Its Metabolic and Pharmaco-Toxicological Implications. Int. J. Mol. Sci..

[32] Whiteley C.G. (2000). Mechanistic and kinetic studies of inhibition of enzymes. Cell Biochem. Biophys..

[33] Chen S., Tang S., Wang Y., Yang Z. (2020). Effect of Astragalus Polysaccharides on Postprandial 1 Hour Blood Glucose in Type 2 Diabetic Rats. Tradit. Chin. Drug Res. Clin. Pharmacol..

[34] Liu Y., Nyberg N.T., Jäger A.K., Staerk D. (2017). Facilitated Visual Interpretation of Scores in Principal Component Analysis by Bioactivity-Labeling of 1H-NMR Spectra-Metabolomics Investigation and Identification of a New α-Glucosidase Inhibitor in Radix Astragali. Molecules.

[35] Zhu Z.Y., Zhang J.Y., Chen L.J., Liu X.C., Liu Y., Wang W.X., Zhang Y.M. (2014). Comparative evaluation of polysaccharides isolated from Astragalus, oyster mushroom, and yacon as inhibitors of α-glucosidase. Chin. J. Nat. Med..

[36] Al-Hmadi H.B., Majdoub S., El Mokni R., Angeloni S., Mustafa A.M., Caprioli G., Zengin G., Maggi F., Hammami S. (2024). Metabolite profiling, enzyme inhibitory activity and antioxidant potential of different extracts from Glycyrrhiza foetida Desf. (Fabaceae, Galegeae, Glycyrrhizinae). Fitoterapia.

[37] Yang L., Jiang Y., Zhang Z.X., Hou J.M., Tian S.K., Liu Y. (2020). The anti-diabetic activity of licorice, a widely used Chinese herb. J. Ethnopharmacol..

[38] Zhang W., Li T., Zhang X.J., Zhu Z.Y. (2020). Hypoglycemic effect of glycyrrhizic acid, a natural non-carbohydrate sweetener, on streptozotocin-induced diabetic mice. Food Funct..

[39] Jia W.J., Bi Q.M., Jiang S.R., Tao J.H., Liu L.Y., Yue H.L., Zhao X.H. (2022). Hypoglycemic activity of *Codonopsis pilosula* (Franch.) Nannf. in vitro and in vivo and its chemical composition identification by UPLC-Triple-TOF-MS/MS. Food Funct..

[40] Su X., Luo L. (2024). UPLC-QE-MS combined network pharmacological to analyze the mechanism and experimental verification of BuzhongYiqi pill in the treatment of ulcerative colitis. Chin. J. Clin. Pharmacol. Ther..

[41] Yan J.K., Chen T.T., Wang L., Wang Z.W., Li C., Chen W.Y., Liu C.H., Li L. (2022). In vitro simulated digestion affecting physicochemical characteristics and bioactivities of polysaccharides from barley (*Hordeum vulgare* L.) grasses at different growth stages. Int. J. Biol. Macromol..

[42] Fang C., Chen G., Kan J. (2022). Characterization and in vitro simulated gastrointestinal digestion and fermentation of Mentha haplocalyx polysaccharide. Int. J. Biol. Macromol..

[43] Zhang G., Yan X., Wu S., Ma M., Yu P., Gong D., Deng S., Zeng Z. (2020). Ethanol extracts from *Cinnamomum camphora* seed kernel: Potential bioactivities as affected by alkaline hydrolysis and simulated gastrointestinal digestion. Food Res. Int..

[44] Tarko T., Duda-Chodak A., Soszka A. (2020). Changes in Phenolic Compounds and Antioxidant Activity of Fruit Musts and Fruit Wines during Simulated Digestion. Molecules.

[45] Martinez-Gonzalez A.I., Díaz-Sánchez A.G., de la Rosa L.A., Vargas-Requena C.L., Bustos-Jaimes I., Alvarez-Parrilla E. (2017). Polyphenolic Compounds and Digestive Enzymes: In Vitro Non-Covalent Interactions. Molecules.

[46] Lam T.P., Tran N.N., Pham L.D., Lai N.V., Dang B.N., Truong N.N., Nguyen-Vo S.K., Hoang T.L., Mai T.T., Tran T.D. (2024). Flavonoids as dual-target inhibitors against α-glucosidase and α-amylase: A systematic review of in vitro studies. Nat. Prod. Bioprospect..

[47] Yang J., Li H., Wang X., Zhang C., Feng G., Peng X. (2021). Inhibition Mechanism of α-Amylase/α-Glucosidase by Silibinin, Its Synergism with Acarbose, and the Effect of Milk Proteins. J. Agric. Food Chem..

[48] Zhao Y., Kongstad K.T., Jäger A.K., Nielsen J., Staerk D. (2018). Quadruple high-resolution α-glucosidase/α-amylase/PTP1B/radical scavenging profiling combined with high-performance liquid chromatography-high-resolution mass spectrometry-solid-phase extraction-nuclear magnetic resonance spectroscopy for identification of antidiabetic constituents in crude root bark of *Morus alba* L. J. Chromatogr. A.

[49] Chen W., Gong L., Guo Z., Wang W., Zhang H., Liu X., Yu S., Xiong L., Luo J. (2013). A novel integrated method for large-scale detection, identification, and quantification of widely targeted metabolites: Application in the study of rice metabolomics. Mol. Plant.

[50] Zhang J., Gong Z., Wang W., Jia F., Cui W., Wang Y. (2017). Method for Detecting Polysaccharide in Water Extraction from Flammulina Velutipes. Farm Prod. Process..

[51] Shen Y., Jin L., Xiao P., Lu Y., Bao J.S. (2009). Total phenolics, flavonoids, antioxidant capacity in rice grain and their relations to grain color, size and weight. J. Cereal Sci..

[52] Singleton V.L. (1999). Analysis of total phenols and other oxidation substrates and their antioxidants by means of Folin-Ciocalteu reagent. Methods Enzymol..

[53] Hadidi M., Ibarz A., Pagan J. (2020). Optimisation and kinetic study of the ultrasonic-assisted extraction of total saponins from alfalfa (*Medicago sativa*) and its bioaccessibility using the response surface methodology. Food Chem..

[54] Luo M.J., Wang Y., Chen S.Y., Yang Z.M. (2022). Astragalus Polysaccharides Alleviate Type 2 Diabetic Rats by Reversing the Expressions of Sweet Taste Receptors and Genes Related to Glycolipid Metabolism in Liver. Front. Pharmacol..

[55] Zhuo J., Zeng Q., Cai D., Zeng X., Chen Y., Gan H., Huang X., Yao N., Huang D., Zhang C. (2018). Evaluation of type 2 diabetic mellitus animal models via interactions between insulin and mitogen-activated protein kinase signaling pathways induced by a high fat and sugar diet and streptozotocin. Mol. Med. Rep..

[56] Mandel A.L., Breslin P.A. (2012). High endogenous salivary amylase activity is associated with improved glycemic homeostasis following starch ingestion in adults. J. Nutr..

[57] Dong Y.X., Li T.H., Wang S.S., Hu Y.H., Liu Y., Zhang F., Sun T.S., Zhang C.J., Du Q.H., Li W.H. (2024). Bu zhong Yiqi Decoction ameliorates mild cognitive impairment by improving mitochondrial oxidative stress damage via the SIRT3/MnSOD/OGG1 pathway. J. Ethnopharmacol..

[58] Zheng Y., Tian J., Yang W., Chen S., Liu D., Fang H., Zhang H., Ye X. (2020). Inhibition mechanism of ferulic acid against α-amylase and α-glucosidase. Food Chem..

[59] Brodkorb A., Egger L., Alminger M., Alvito P., Assunção R., Ballance S., Bohn T., Bourlieu-Lacanal C., Boutrou R., Carrière F. (2019). INFOGEST static in vitro simulation of gastrointestinal food digestion. Nat. Protoc..

[60] Yao X., Zhu L., Chen Y., Tian J., Wang Y. (2013). In vivo and in vitro antioxidant activity and α-glucosidase, α-amylase inhibitory effects of flavonoids from *Cichorium glandulosum* seeds. Food Chem..

