# Supplementary material for: BuZhong YiQi Formula Alleviates Postprandial Hyperglycemia in T2DM Rats by Inhibiting α-Amylase and α-Glucosidase In Vitro and In Vivo
Source: Pharmaceuticals (Basel). 2025 Feb 2;18(2):201. doi: 10.3390/ph18020201 (PMC11858844; doi:10.3390/ph18020201)
Supplement: Supplementary file 1 [file pharmaceuticals-18-00201-s001.zip › Content of calycosin-7-O-β-D-glucoside in BZYQF.pdf]

Chromatogram and Results

Injection Details

Injection Name:	**样成80**	*Run Time (min):*	**78.00**
Vial Number:	**GE2**	*Injection Volume:*	**20.00**
Injection Type:	**Unknown**	*Channel:*	**UV_VIS_1**
Calibration Level:		*Wavelength:*	**214**
Instrument Method:	样品方法	*Bandwidth:*	**4**
Processing Method:	**Basic Quantitative(2)**	*Dilution Factor:*	**1.0000**
Injection Date/Time:	**03/四月/24 12:35**	*Sample Weight:*	**1.0000**

Chromatogram

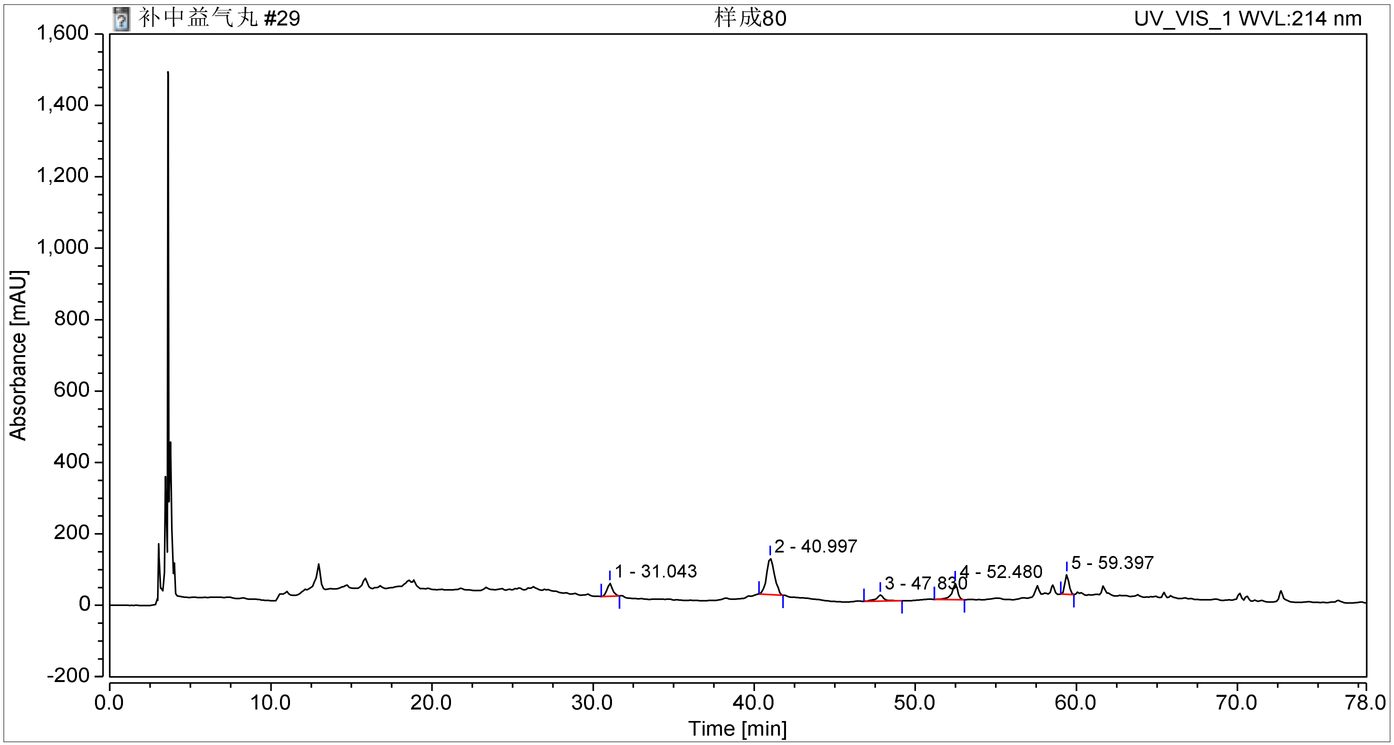

Integration Results

No.	Peak Name	Retention Time min	Area mAU*min	Height mAU	Relative Area %	Relative Height %	Amount n.a.
1		31.043	14.298	35.774	12.11	14.05	n.a.
2		40.997	58.482	101.366	49.55	39.82	n.a.
3		47.830	9.494	16.981	8.04	6.67	n.a.
4		52.480	19.282	45.077	16.34	17.71	n.a.
5		59.397	16.471	55.333	13.96	21.74	n.a.
**Total:**			**118.028**	**254.531**	**100.00**	**100.00**	

Chromeleon (c) Dionex
Version 7.2.9.11323
