# Supplementary material for: BuZhong YiQi Formula Alleviates Postprandial Hyperglycemia in T2DM Rats by Inhibiting α-Amylase and α-Glucosidase In Vitro and In Vivo
Source: Pharmaceuticals (Basel). 2025 Feb 2;18(2):201. doi: 10.3390/ph18020201 (PMC11858844; doi:10.3390/ph18020201)
Supplement: Supplementary file 1 [file pharmaceuticals-18-00201-s001.zip › Content of Standard (calycosin-7-O-β-D-glucoside).pdf]

Chromatogram and Results

Injection Details

Injection Name:	标品	*Run Time (min):*	**65.00**
Vial Number:	**GE1**	*Injection Volume:*	**20.00**
Injection Type:	**Unknown**	*Channel:*	**UV_VIS_1**
Calibration Level:		*Wavelength:*	**223**
Instrument Method:	标品方法	*Bandwidth:*	**4**
Processing Method:	**Basic Quantitative(2)**	*Dilution Factor:*	**1.0000**
Injection Date/Time:	**07/三月/24 15:43**	*Sample Weight:*	**1.0000**

Chromatogram

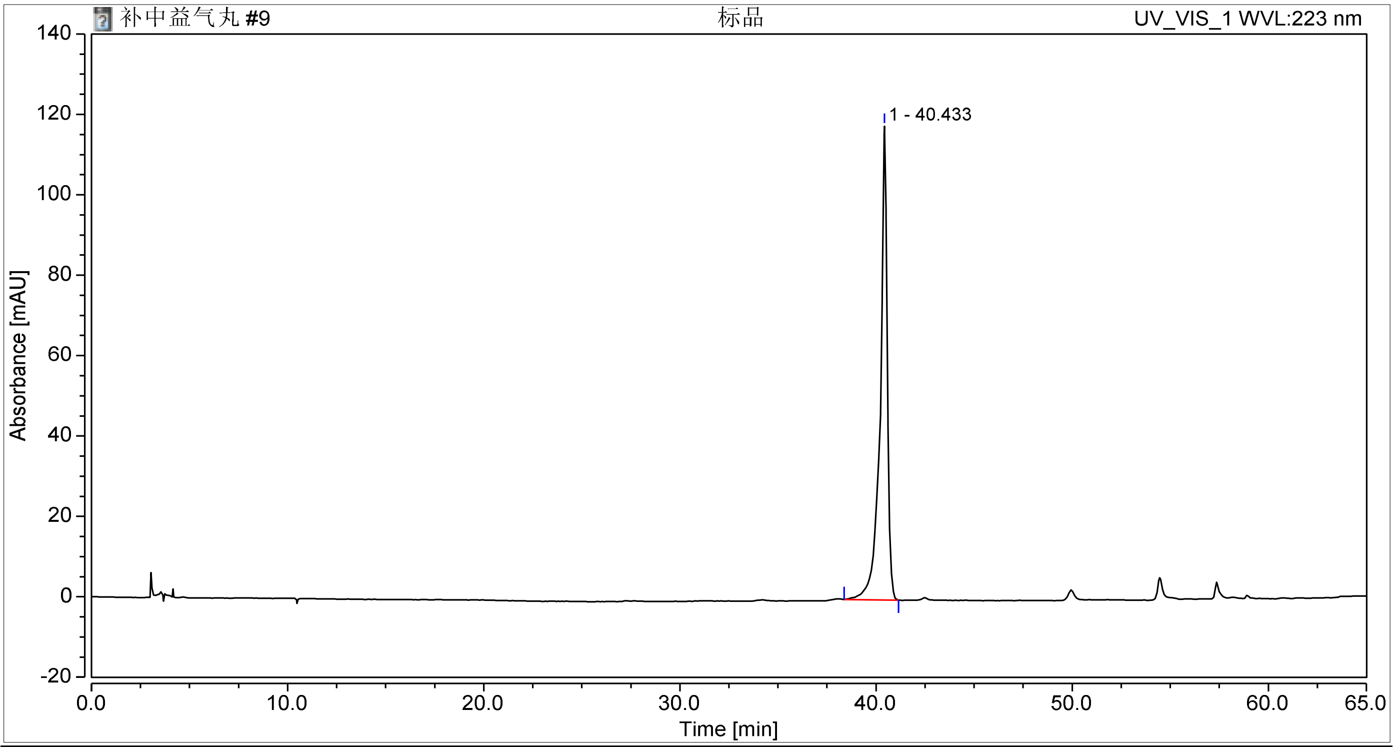

Integration Results

No.	Peak Name	Retention Time min	Area mAU*min	Height mAU	Relative Area %	Relative Height %	Amount n.a.
1		40.433	48.770	117.847	100.00	100.00	n.a.
**Total:**			**48.770**	**117.847**	**100.00**	**100.00**	
