# Supplementary figures and images for: BuZhong YiQi Formula Alleviates Postprandial Hyperglycemia in T2DM Rats by Inhibiting α-Amylase and α-Glucosidase In Vitro and In Vivo
Source: Pharmaceuticals (Basel). 2025 Feb 2;18(2):201. doi: 10.3390/ph18020201 (PMC11858844; doi:10.3390/ph18020201)

■ XIC of -MRM (1906 pairs): 198.000/181.000 amu Expected RT: 2.6 ID: mws0923\_N from Sample 2 (T2360676a\_N) of MWXS-23-777-01-a\_1\_W...

Max. 2.9e5 cps.

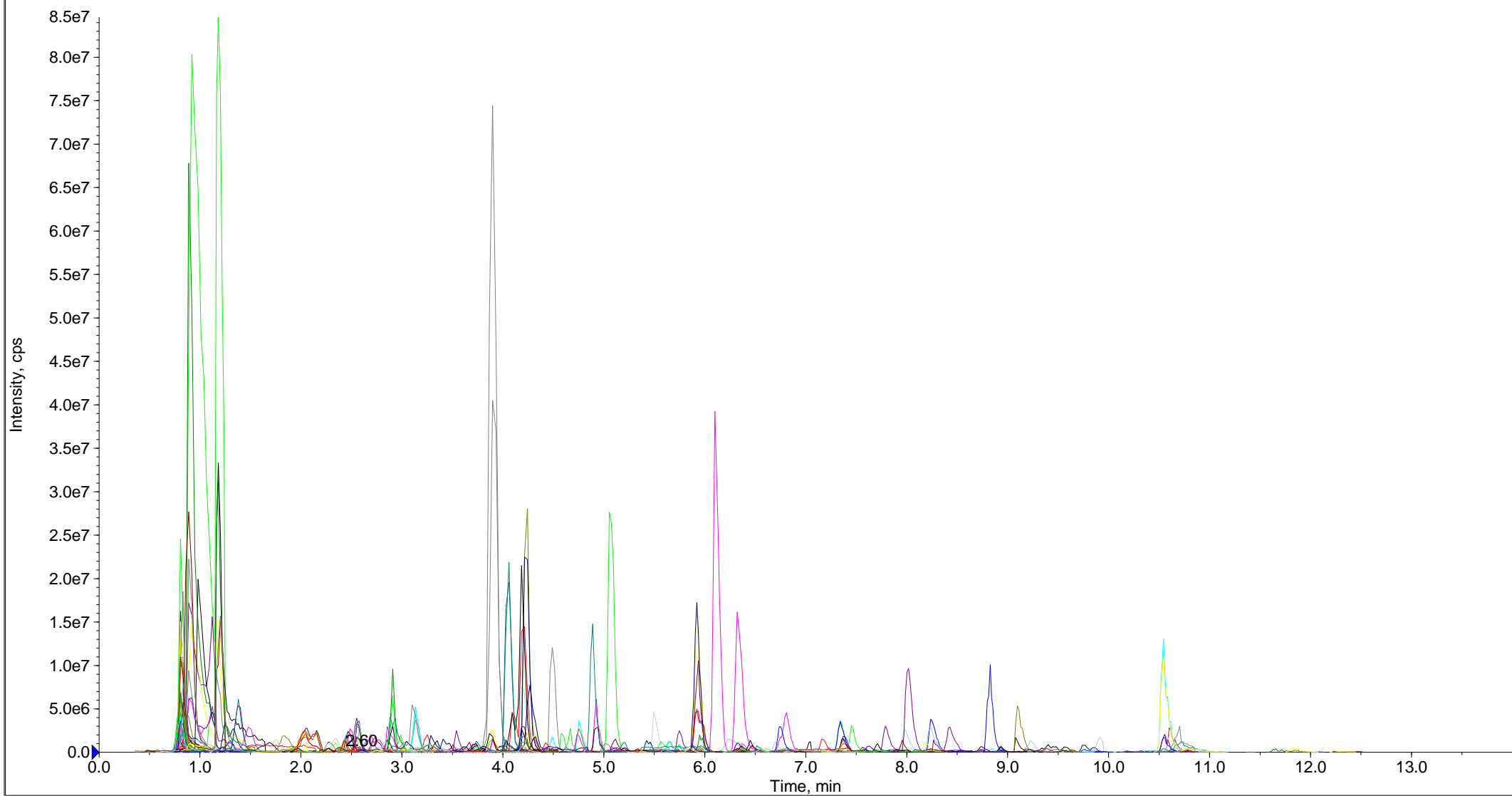

Supplement: Supplementary file 1 [file pharmaceuticals-18-00201-s001.zip › MRM_detection_of_multimodal_maps-N.pdf]

■ XIC of +MRM (2237 pairs): 200.000/154.000 amu Expected RT: 2.5 ID: mws0923\_P from Sample 1 (T2360676a\_P) of MWXS-23-777-01-a\_1\_W...

Max. 1.2e7 cps.

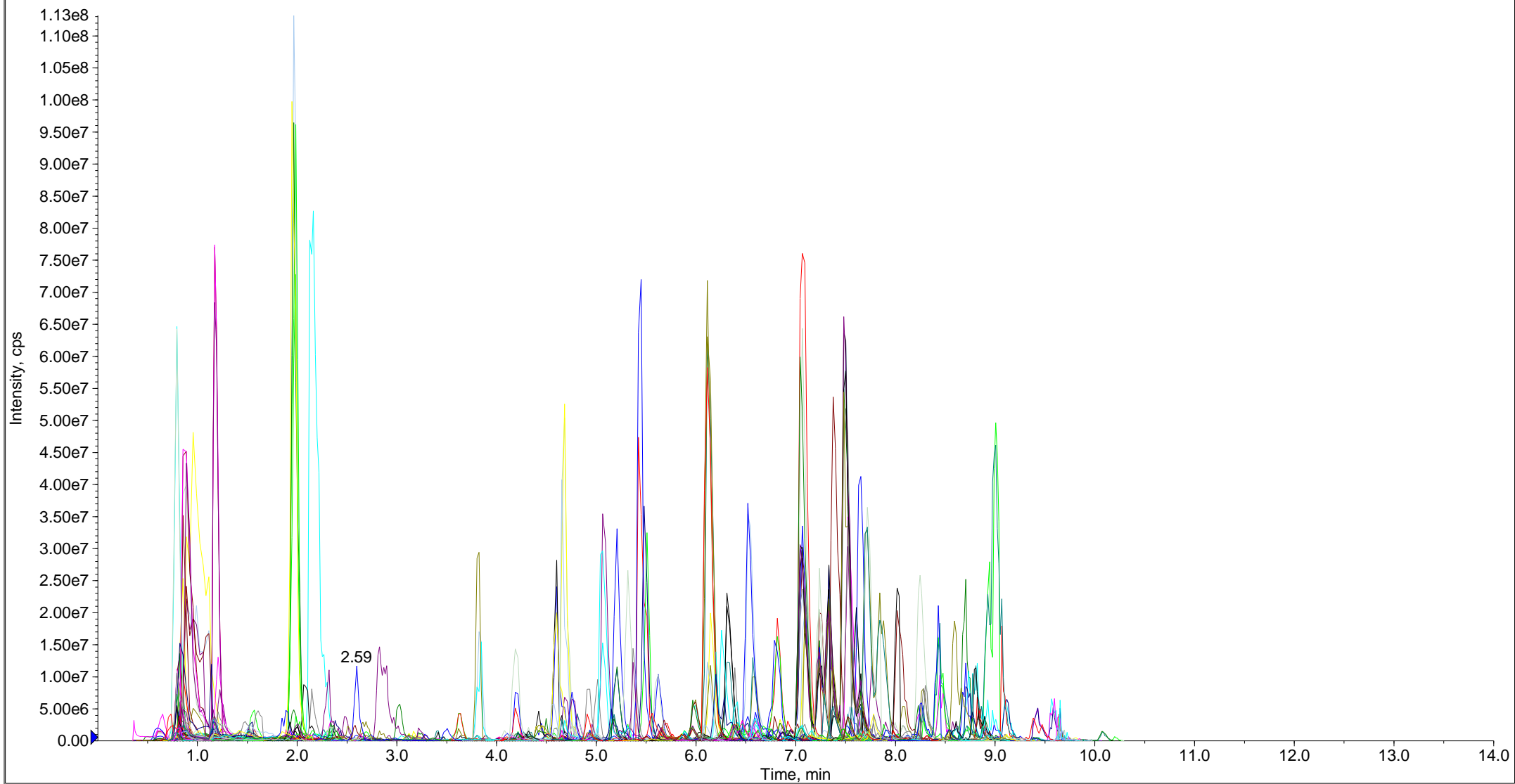

Supplement: Supplementary file 1 [file pharmaceuticals-18-00201-s001.zip › MRM_detection_of_multimodal_maps-P.pdf]

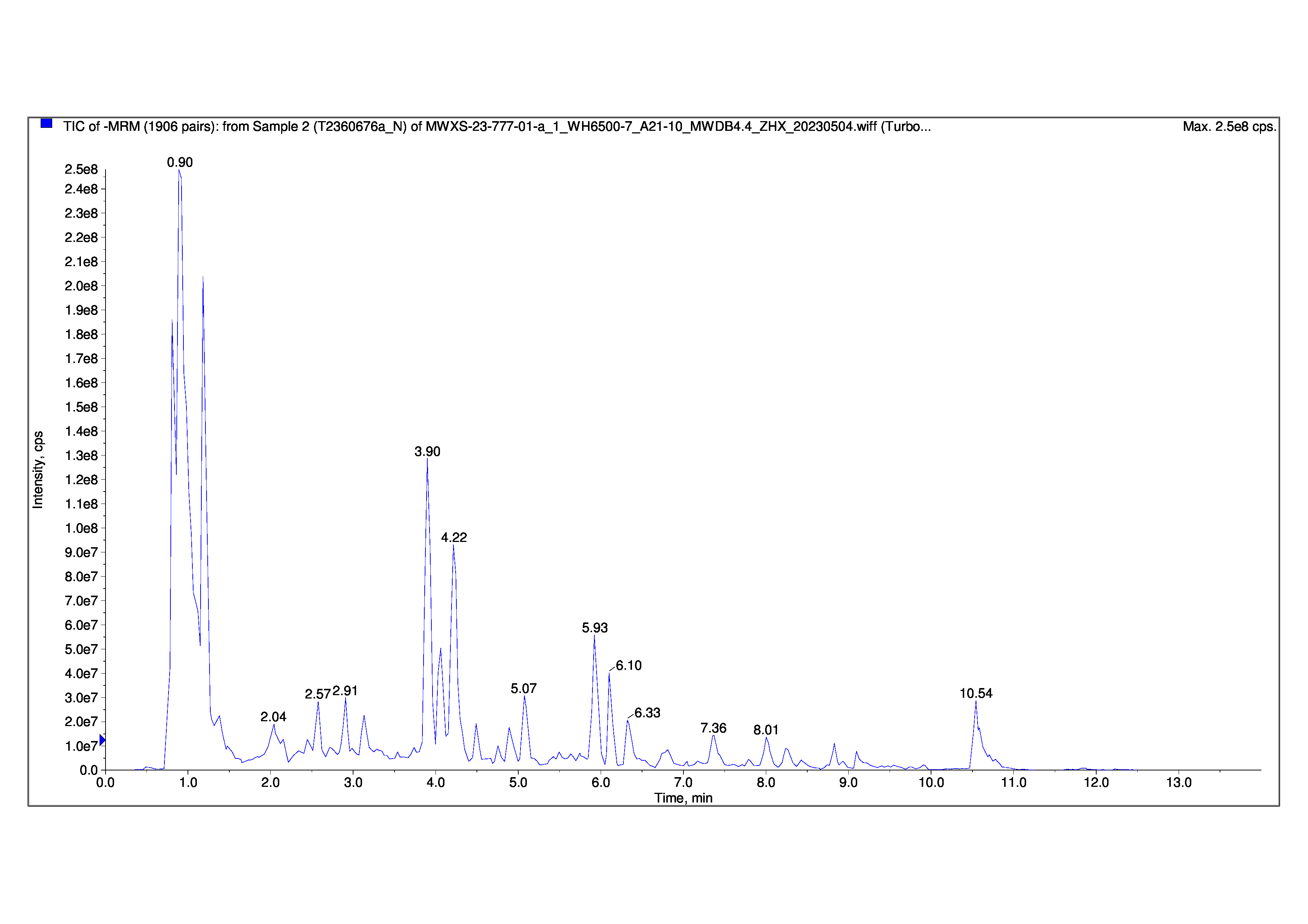

Supplement: Supplementary file 1 [file pharmaceuticals-18-00201-s001.zip › MWXS-23-777-01-a_QC_MS_TIC-N.png]

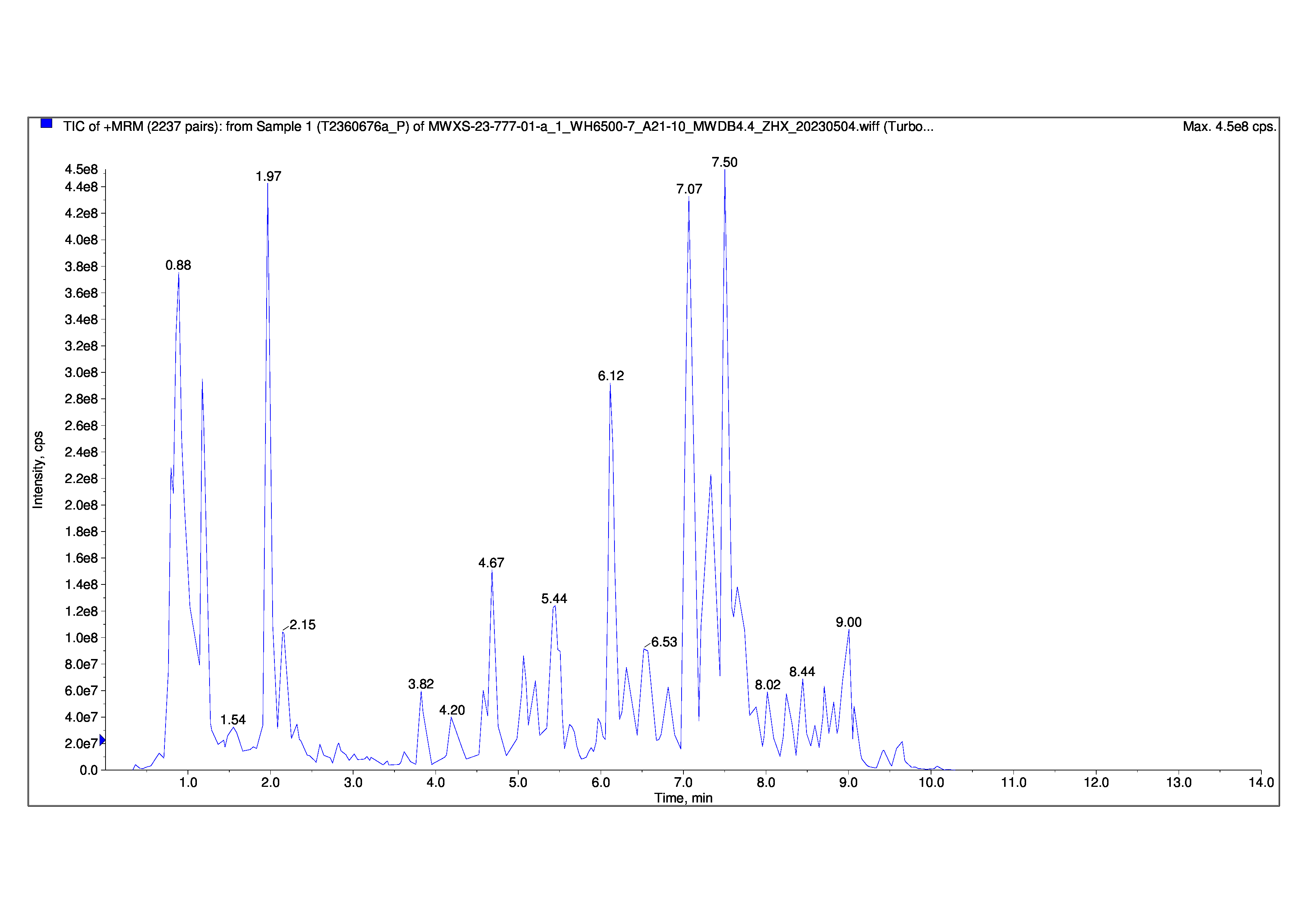

Supplement: Supplementary file 1 [file pharmaceuticals-18-00201-s001.zip › MWXS-23-777-01-a_QC_MS_TIC-P.png]
